# Pinacidil ameliorates cardiac microvascular ischemia–reperfusion injury by inhibiting chaperone-mediated autophagy of calreticulin

**DOI:** 10.1007/s00395-023-01028-8

**Published:** 2024-01-02

**Authors:** Muyin Liu, Su Li, Ming Yin, Youran Li, Jinxiang Chen, Yuqiong Chen, You Zhou, Qiyu Li, Fei Xu, Chunfeng Dai, Yan Xia, Ao Chen, Danbo Lu, Zhangwei Chen, Juying Qian, Junbo Ge

**Affiliations:** 1 Department of Cardiology, Zhongshan Hospital, Fudan University, Shanghai, 200032 China; 2 Shanghai Institute of Cardiovascular Diseases, Shanghai, 200032 China; 3 National Clinical Research Center for Interventional Medicine, Shanghai, 200032 China; 4 Gusu School, The Affiliated Suzhou Hospital of Nanjing Medical University, Suzhou Municipal Hospital, Nanjing Medical University, Nanjing, China; 5 Department of Cardiology, West China Hospital, Sichuan University, Chengdu, People’s Republic of China

**Keywords:** Cardiac microvascular ischemia–reperfusion injury, Pinacidil, Calreticulin, Chaperone-mediated autophagy, Calcium overload, Mitochondrial injury

## Abstract

Calcium overload is the key trigger in cardiac microvascular ischemia–reperfusion (I/R) injury, and calreticulin (CRT) is a calcium buffering protein located in the endoplasmic reticulum (ER). Additionally, the role of pinacidil, an antihypertensive drug, in protecting cardiac microcirculation against I/R injury has not been investigated. Hence, this study aimed to explore the benefits of pinacidil on cardiac microvascular I/R injury with a focus on endothelial calcium homeostasis and CRT signaling. Cardiac vascular perfusion and no-reflow area were assessed using FITC–lectin perfusion assay and Thioflavin-S staining. Endothelial calcium homeostasis, CRT–IP3Rs–MCU signaling expression, and apoptosis were assessed by real-time calcium signal reporter GCaMP8, western blotting, and fluorescence staining. Drug affinity-responsive target stability (DARTS) assay was adopted to detect proteins that directly bind to pinacidil. The present study found pinacidil treatment improved capillary density and perfusion, reduced no-reflow and infraction areas, and improved cardiac function and hemodynamics after I/R injury. These benefits were attributed to the ability of pinacidil to alleviate calcium overload and mitochondria-dependent apoptosis in cardiac microvascular endothelial cells (CMECs). Moreover, the DARTS assay showed that pinacidil directly binds to HSP90, through which it inhibits chaperone-mediated autophagy (CMA) degradation of CRT. CRT overexpression inhibited IP3Rs and MCU expression, reduced mitochondrial calcium inflow and mitochondrial injury, and suppressed endothelial apoptosis. Importantly, endothelial-specific overexpression of CRT shared similar benefits with pinacidil on cardiovascular protection against I/R injury. In conclusion, our data indicate that pinacidil attenuated microvascular I/R injury potentially through improving CRT degradation and endothelial calcium overload.

**Supplementary Information:**

The online version contains supplementary material available at 10.1007/s00395-023-01028-8.

## Introduction

Cardiac microvascular dysfunction occurs in a sizeable proportion of patients after percutaneous coronary intervention (PCI), leading to ineffective myocardial reperfusion, exacerbating myocardial damage, and contributing to major adverse cardiovascular events [43]. In addition, together with the rupture of the atherosclerotic plaques and even the plaque materials released during PCI, microembolism of the cardiac microcirculation is frequently identified [25]. Therefore, the significance of cardiac microcirculation in safeguarding cardiac function has garnered increasing attention [21]. Accumulated evidence has shown that cardiac microvascular protection strategies can reduce myocardial edema and leukocyte adherence, improve myocardial perfusion, minimize the extent of the no-reflow area, and increase the life expectancy of patients following PCI [19, 20, 34]. Therefore, coronary microcirculation is a valid target for cardioprotection after acute myocardial ischemia–reperfusion (I/R) injury [17]. Hence, additional research is necessary to fully understand the underlying mechanisms of cardiac microvascular dysfunction to formulate more effective therapeutic strategies.

Mitochondrial dysfunction is a well-established hallmark of cardiac microvascular endothelial cells (CMECs) injury, which activates programmed cell death and ultimately leads to cardiac microvascular dysfunction [8, 35, 56]. Calcium overload promotes mitochondrial dysfunction and is involved in cardiovascular diseases, including I/R injury, hypertension, and heart failure [7, 34]. The maintenance of calcium homeostasis is primarily regulated by various membrane calcium channels, such as inositol 1,4,5-triphosphate receptors (IP3Rs), ATPase sarcoplasmic/endoplasmic reticulum calcium transporting 2 (SERCA2), and mitochondrial calcium uniporter (MCU), which are mainly located in the endoplasmic reticulum (ER)–mitochondria coupling site, known as mitochondria-associated membranes (MAMs) [44, 61]. Specifically, IP3Rs are localized on the ER membrane and release calcium from ER hotspots to the mitochondria [5]. MCU is located in the mitochondrial outer membrane and mediates calcium influx into mitochondria, resulting in mitochondrial calcium overload [33, 62]. In contrast, SERCA2, a calcium tunnel on the ER membrane reduces mitochondrial calcium levels. Hypoxia/reoxygenation injury increases the expression of IP3Rs and MCU, causing a rapid increase in calcium levels, which subsequently leads to endothelial apoptosis [65]. On the contrary, inhibiting IP3Rs and MCU in the endothelium can restrain mitochondrial calcium uptake, alleviate cytoplasmic and mitochondrial calcium overload, and ultimately improve cardiac microvascular I/R injury [55, 67].

Calreticulin (CRT) is a calcium-binding protein located in the ER with a high affinity and capacity for calcium buffering [12]. An increased abundance of CRT can enhance ER calcium stores and thereby support calcium homeostatic recovery. Furthermore, accumulating evidence indicates diverse non-ER functions for CRT, including integrin adhesion, fibroblast migration, macrophage-mediated phagocytosis, and immunoregulatory functions [16]. Similarly, the role of CRT in cardiac endothelial protection has been discussed since it promotes VEGF-A mRNA stabilization and thereby angiogenesis in gastric cancer [32]; stabilizes F-actin via actin acetylation; protects microvascular endothelial cells against microwave radiation [57]; stimulates endothelial nitric oxide (NO) production and limits thrombosis in canine coronary arteries [30]. Importantly, CRT ameliorates oxygen glucose deprivation/reoxygenation (OGD/R)-induced microvascular endothelial cells injury by inhibiting autophagy [58]. In contrast, some studies have reported that CRT and its fragments, vasostatins, inhibit angiogenesis and suppress tumor growth [45]. These results suggest that the effects of CRT on endothelial protection and angiogenesis may be context dependent. Moreover, the role of CRT in cardiac microvascular I/R injury has not yet been explored and requires further research to understand its potential implications.

Pinacidil belongs to the family of nonselective ATP-sensitive K (K_ATP_) channel openers and is clinically used as an antihypertensive drug due to its ability to directly relax vascular smooth muscle cells (VSMCs) promoting peripheral vasodilation [10, 11]. In addition, pharmacological pre-conditioning or post-conditioning with pinacidil can improve the coronary vasodilatory effects and protect the ischemic heart from reperfusion injury [48, 64]. However, except its ability to relax VSMCs, the potential of pinacidil on endothelium and endothelial cells in cardiovascular injury has not been investigated. Only limited evidence suggested pinacidil enhances the activity of endothelial NO during OGD/R injury [51]. Considering endothelial cells are the anatomical and functional basis of cardiac microvasculature, it is relevant and essential to discuss the role of K_ATP_ channel and pinacidil in endothelial and cardiovascular protection.

To address these issues, the present study was conducted to investigate the beneficial effects of pinacidil on cardiac microvascular protection after I/R injury, with an emphasis on endothelial calcium channel modulation and mitochondrial dysfunction.

## Method

### Animals and treatment

All animal experiments and procedures were approved by the Institutional Animal Care and Use Committee of Zhongshan Hospital, Fudan University, Shanghai, China (approval number: 2019–309). Male C57BL/6 J mice aged 6–8 weeks were procured from Shanghai SLAC Laboratory Animal Co., Ltd. (Shanghai, China) and housed in a specific pathogen-free room.

CRT-overexpressing adeno-associated virus serotype 9 (AAV9-CRT) genomic particles were obtained from Genomeditech (Shanghai, China). To construct an endothelial cell-specific CRT overexpression mouse model, 5 × 10^11^ AAV9-CRT or its negative control (AAV9-NC) genome particles was injected into 6-week-old C57BL/6 J mice via the caudal vein. After 4 weeks, the transfection efficiencies of AAV9-CRT and AAV9-NC in the left ventricle (LV) tissues were determined by immunofluorescence co-staining with Flag and CD31. CRT overexpression efficiency was determined by western blotting analysis of primary mouse CMECs (MCMECs).

The I/R injury was induced in mice by ligating the left anterior descending coronary artery (LAD) using a slipknot with 6-0 silk for 45 min, followed by loosening to induce reperfusion injury, as previously described [34]. Pinacidil (MCE, USA) was injected intraperitoneally at doses of 0.1 or 0.5 mg/kg/day for 3 days before the induction of I/R injury [50]. Three days after I/R injury, cardiac function was measured by echocardiography using a VisualSonics Vevo770 (VisualSonics, Toronto, Canada).

### Hemodynamic analysis

Hemodynamic analysis was performed by pressure–volume analysis via the Transonic Pressure–Volume Measurement System (Scisense, Shanghai). After I/R injury and treatment, inhalant anesthetic was used before establishing a temporary airway via tracheotomy to perform the PV-loop analysis. A 1.2F catheter transducer (Scisense, Shanghai) was carefully placed close to the center of the apex in the LV through a right carotid artery cannulation. The heart rate (HR), end-systolic pressure–volume relationship (ESPVR), end-diastolic pressure–volume relationship (EDPVR), left ventricle end-diastolic pressure (LVEDP), + dP/dt(mmHg/s), and -dP/dt (mmHg/s) were measured through the catheter and Transonic Pressure–Volume Measurement System [53].

### Histopathological examination

Cardiac tissues were fixed with 10% formalin solution, gradually dehydrated, embedded in paraffin, and then cut into 4-μm-thick sections. Interstitial fibrosis was assessed by Masson’s trichrome staining. The percentage of interstitial fibrosis was calculated using the ImageJ software (Version 1.8, National Institute of Health (NIH), Bethesda, MD, USA). For immunohistochemical staining, a primary anti-CRT antibody (1:200, ab92516; Abcam, Cambridge, UK) was used. At least five randomly selected microscopic fields (magnification, 200 ×) were captured.

### Evans Blue/2,3,5-triphenyl tetrazolium chloride (TTC) staining

TTC staining was performed 3 days after surgery on the sham group, I/R injury group, and I/R + pinacidil groups (0.1 and 0.5 mg/kg/day) to measure the benefits of pinacidil on infraction size according to previous guideline [4]. Similarly, TTC staining was also performed on the sham and I/R groups that were transfected with or without AAV9-CRT. After the mice were anesthetized with isoflurane vapor, the LAD was ligated again at the same level and 1% Evans blue was injected through the LV outflow tract. Each heart was immediately excised and cut evenly into 1-mm-thick slices from the apex to the bottom on frozen ice. The slices were incubated in 1% TTC solution at 37 ℃ for 10 min and fixed in 4% paraformaldehyde (PFA) overnight. The samples were then scanned using a white-light scanner (Canon, Tokyo, Japan). The area at risk (AAR) and infarct area of each slice were measured using the ImageJ software (Version 1.8, NIH). The relative infarct size was calculated as the ratio of the infarct area to the AAR.

### Thioflavin-S staining

Thioflavin-S staining was conducted 3 days after surgery on the sham group, I/R injury group, and I/R + pinacidil groups (0.1 and 0.5 mg/kg/day) to measure the benefits of pinacidil on no-reflow area according to previous study [29]. Similarly, Thioflavin-S staining was also performed on the sham and I/R groups that were transfected with or without AAV9-CRT. 2% thioflavin-S (MCE, USA) was dissolved in normal saline and injected into a different set of mice via the tail vein. After ten cardiac cycles, mouse hearts were immediately harvested, quickly washed with cold PBS, and fixed in 4% PFA overnight. The heart was cut into 1 mm slices, exposed to ultraviolet light, and photographed by a stereomicroscope (Leica, Germany). The no-reflow degree was calculated by the ratio of the dark area to the LV area.

### Cardiac vascular perfusion detection

100 μl FITC–lectin (Sigma, St. Louis, MO, USA) at a concentration of 1 mg/mL was injected into the mice via the caudal vein to label the perfused vessels [39]. Ten minutes after injection, the heart samples were harvested to prepare frozen sections and immunostained with CD31. The vascular perfusion was determined by the ratio of FITC–labeled vessels to CD31-stained vessels.

### Calcium signal in cardiac microcirculation

Real-time recording of cardiac microvascular calcium signal was identified by GCaMP8 [9]. Cdh5-GCaMP8 overexpressing adeno-associated virus serotype 9 (AAV9-Cdh5-GCaMP8) genomic particles were obtained from HANBIO (Shanghai, China). 5 × 10^11^ AAV9-Cdh5-GCaMP8 genome particles were injected into the 6-week-old C57BL/6 J mice via the tail vein. After 4 weeks, the transfection efficiencies in the LV tissues were determined using immunofluorescence.

### Myocardial water content measurement

Cardiac water content was evaluated using the wet/dry weight ratio method at the end of treatment [18]. Wet weight of LV was measured immediately after killing, and the dry weight was obtained after desiccation for 5 days at 65 °C. Myocardial water content was calculated as: water content = (wet heart weight—dry heart weight)/wet heart weight × 100%.

### CMA activation in cardiac microcirculation

The CMA activation was detected via mCherry-KFERQ reporter [60]. mCherry-KFERQ-NE overexpressing adeno-associated virus serotype 9 (AAV9-mCherry-KFERQ-NE) genomic particles with Cdh5 promoter were constructed, and 5 × 10^11^ AAV9-mCherry-KFERQ-NE genome particles were injected into 6-week-old C57BL/6 J mice via the tail vein [40]. After I/R injury and pinacidil treatment, the primary MCMECs were extracted from the LV, and the red CMA puncta dots were observed and calculated under a confocal microscope (Olympus FV3000, Tokyo, Japan).

### Cell isolation, culture, and treatment

Primary MCMECs were extracted from the LV of C57BL/6 mice according to a previously reported method [24]. The endocardium, epicardium, and coronary arteries were excised first and then the LV tissue was minced and digested with liberase. The cell suspension was incubated with anti-CD31 antibody (Abcam, ab7388)-conjugated microbeads (Thermo Fisher Scientific, Waltham, MA, USA) for 30 min at 4 °C under slow and constant rotation. Subsequently, primary MCMECs were collected using a magnetic separator and cultured in fibronectin-coated dishes with complete endothelial culture medium (ECM; ScienCell, Carlsbad, CA, USA). Human CMECs (HCMECs) at passage 3 were purchased from Lonza Bioscience (Basel, Switzerland) and cultured in ECM.

To induce OGD/R injury, the HCMECs were cultured in Eagle’s solution (Genom, Hangzhou, China) and maintained in a hypoxic chamber (5% CO_2_ and 95% N_2_, 37 °C) for 12 h [66, 68]. Subsequently, the culture conditions were restored to normal atmosphere and ECM to induce reoxygenation injury. Cells in the pinacidil-treated groups were incubated with 0.1 or 10 μM pinacidil [64]. Cell viability was detected using a Cell Counting Kit-8 (CCK8) assay kit (EpiZyme, Shanghai, China) according to the manufacturer's instructions.

### Chemical reagents

Chloroquine (CQ) and bafilomycin A1 (Baf A1) were purchased from Selleck Chemicals (Houston, TX, USA). MG132 was purchased from Sigma-Aldrich. HCMECs were pretreated with MG132 (10 μM), CQ (20 μM), and Baf A1 (500 ng/mL) for 2 h before OGD/R injury treatment.

### Overexpression and knockdown of CRT

Lentiviruses encoding the CRT sequence (LV-CRT), short hairpin-CRT (LV-shCRT), or negative control (LV-NC) were purchased from Genomeditech (Shanghai, China). HCMECs were transfected with LV-CRT, LV-shCRT, and LV-NC at multiplicities of infection (MOIs) of 10 according to the manufacturer's instructions. After 48 h transfection, the stably transfected cell line was selected for using 3 μg/mL puromycin. CRT overexpression and knockdown efficiencies were determined using western blotting.

### Endothelial monolayer permeability assays

HCMECs were seeded in the upper chamber of a Corning Transwell plate (0.4 μm pore size, 6.5 mm diameter; Corning Incorporated, NY, USA). After OGD/R injury and pinacidil treatment, 100 μL of 1 mg/mL FITC–BSA (68 kDa, Sigma, USA) was added to the upper chamber and allowed to permeate freely to the lower chamber for 1 h. The fluorescence intensity of FITC–BSA in both upper and lower chambers was quantified using a FlexStation 3 (Molecular Devices, USA), and the ratio of upper/lower fluorescence intensity was calculated to reflect the permeability of the endothelial monolayer.

### 5-Ethynyl-2′-deoxyuridine (EdU) staining

The EdU assay was conducted using the BeyoClick™ EdU Cell Proliferation Kit with Alexa Fluor 555 (Beyotime Biotechnology, Shanghai, China). Cell nuclei were labeled with 4′,6-diamidino-2-phenylindole (DAPI) reagent. The samples were observed under a confocal microscope (Olympus FV3000, Tokyo, Japan) and analyzed using the ImageJ software (Version 1.8, NIH).

### NO detection

The NO content in the cardiac tissue and cell culture was measured using a nitrate/nitrite assay kit (Beyotime Biotechnology). Cardiac lysates, cell culture medium, and standard substances were incubated with working solutions in the dark as standard protocol, after which the OD values were read at 540 nm by FlexStation 3 (Molecular Devices, USA). The protein concentration of the cardiac lysate was assessed using a Bradford protein assay kit (Solarbio, Beijing, China), and the NO content in the cardiac lysate was standardized using protein concentration. The NO content in the HCMECs was directly measured using a Micro NO Content assay kit (Solarbio).

### Reactive oxygen species (ROS) and calcium overload measurement

Oxidant stress injury was evaluated by measuring the production of intracellular ROS and mitochondrial ROS (mtROS) by DCFH-DA (Beyotime Biotechnology) and MitoSOX™ Red Mitochondrial Superoxide Indicator (Invitrogen, Carlsbad, CA, USA), respectively. Cells after treatment were incubated with 10 μM DCFH-DA or 1 μM MitoSOX in fetal bovine serum (FBS)-free medium for 20–30 min at 37 °C, and washed with PBS thrice. Intracellular calcium [Ca^2+^]_i_ overload and mitochondrial calcium [Ca^2+^]_m_ levels were measured by Fluo-4 and Rhod-2 (Thermo Fisher, USA), respectively. HCMECs after different treatments were incubated with 4 μM Fluo-4 and 1 μM Rhod-2 in FBS-free medium for 30 min in the dark. Immunofluorescence intensity was observed under a confocal microscope (Olympus FV3000) and analyzed using the ImageJ software (Version 1.8, NIH).

### Mitochondrial function measurement

Live mitochondria were labeled by MitoTracker Deep Red^FM^ (Thermo Fisher Scientific), cellular mitochondrial membrane potential was measured by the TMRM Reagent (MedChemExpress, NJ, USA) and ER was detected by ER-Tracker Red (Beyotime Biotechnology). Primary MCMECs and HCMECs were cultured in the confocal dishes and went through different treatment before mitochondrial function detection. Cells were incubated with 5 μM MitoTracker Deep Red^FM^, 2 μM TMRM Reagent, and 1 μM ER-Tracker Red for 20 min in the dark, respectively. The live cell samples were observed and imaged under a confocal microscope (Olympus FV3000, Olympus). Fluorescence intensity was analyzed using the ImageJ software (Version 1.8, NIH), and mitochondrial length was measured using MiNA (https://github.com/ScienceToolkit/MiNA).

### Immunofluorescence staining

Frozen heart sections were fixed with cold acetone for 10 min at – 20 ℃, followed by airing until completely dried. Similarly, cell samples were fixed with 4% PFA for 10 min at 37 ℃ and extensively washed with PBS. Subsequently, both heart sections and cell samples were permeabilized with 0.5% Triton X-100 for 10 min, blocked with 5% BSA for 1 h at room temperature, incubated with primary antibodies at 4 ℃ overnight, and finally stained with fluorescence-labeled secondary antibodies. The detailed information on the antibodies used in immunofluorescence staining can be found in Table S1. All tissue sections and confocal dishes were observed under a confocal microscope (Olympus FV3000). At least 20 random fields were captured from six samples and quantified using the ImageJ software (Version 1.8, NIH).

### Apoptosis assay

Apoptosis in both cell and tissue sections was assessed using the TUNEL assay. Cells were fixed, permeabilized, and incubated with the TUNEL reaction buffer (Sigma-Aldrich) for 60 min. TUNEL staining on frozen sections were performed by the One Step TUNEL Apoptosis Assay Kit (Beyotime Biotechnology). The sections were stained with TUNEL working solution for 120 min and then co-incubated with cTnT and CD31 primary antibodies at 4 ℃ overnight. Then the slides were stained with fluorescence-labeled secondary antibodies and sealed with Antifade Mounting Medium (Beyotime Biotechnology). The samples were observed under a confocal microscope (Olympus FV3000, Japan).

### Co-immunoprecipitation (Co-IP) assay

HCMECs were lysed using IP lysis buffer (Beyotime Biotechnology). The antibodies against CRT (#NBP1-97,502, Novus Biologicals, Littleton, CO, USA), HSP90B (#NB110-61640, Novus Biologicals), or IgG control (#5873, Cell Signaling Technology, Danvers, MA, USA) were pre-incubated with magnetic beads (Biolinkedin, China) at 4 ℃ for 2 h. Then, the cell lysates were incubated with the magnetic beads at 4 ℃ overnight. The next day, after extensive washing, the samples were boiled with loading buffer for 10 min and collected for subsequent western blotting analysis.

### Western blotting assay

Both heart and cell samples were lysed in RIPA lysis buffer containing PMSF and phosphatase inhibitor cocktail A (Beyotime Biotechnology). After centrifugation at 12 000 × *g* for 30 min, the supernatants were collected and quantified using a BCA Protein Assay Kit (Beyotime Biotechnology). Subsequently, the protein samples were loaded onto sodium dodecyl sulfate (SDS)-polyacrylamide gels for electrophoresis and then transferred to polyvinyl difluoride (PVDF) membranes. After blocking in 5% BSA 1 h, the membranes were incubated with primary antibodies at 4 ℃ overnight and horseradish peroxidase (HRP)-conjugated secondary antibodies at room temperature for 1 h. Protein densitometry was performed using Pierce™ Enhanced Chemiluminescence (ECL) Western Blotting Substrate (Thermo Fisher Scientific) and quantified using the ImageJ software (Version 1.8, NIH). The western blotting cuts were originated from one gel run and one membrane when proteins’ molecular weights were able to be separated by standard markers. In addition, the proteins with the same or approximate molecular weights were originally cut from different membranes, but with the same loading quantity. The detailed information on the antibodies used in the western blotting assay is presented in Table S2.

### Wound-healing and tube formation assays

After the cells reached 100% confluence, a wound-healing assay was performed by scratching the cell monolayer with a 200-µL pipette tip, washing with PBS, and replacing the culture medium with ECM without FBS. Images were collected right after and 48 h after scratching. The rate of wound healing was determined by calculating the proportion of the healed areas after 48 h.

To measure angiogenesis in HCMECs, a capillary tube formation assay was performed. Matrigel (50 µL, BD Bioscience, Bedford, MA, UDA) was added to a 96-well plate and solidified at 37 ℃ for 1 h. Subsequently, a cell suspension was seeded onto the Matrigel and incubated for 6 h, after which capillary-like tubes were observed using an optical microscope (Leica DM3000, Wetzlar, Germany), and the number of branch points was counted using the ImageJ software (Version 1.8, NIH).

### RNA-sequencing assay

Total RNA was extracted using the TRIzol reagent (Thermo Fisher Scientific) according to the manufacturer’s protocol. RNA purity and quantity were evaluated using a NanoDrop 2000 spectrophotometer (Thermo Fisher Scientific). RNA integrity was assessed using the Agilent 2100 Bioanalyzer (Agilent Technologies, Santa Clara, CA, USA). Subsequently, libraries were constructed using TruSeq Stranded mRNA LT Sample Prep Kit (Illumina, San Diego, CA, USA), according to the manufacturer’s instructions. Transcriptome sequencing and analysis were performed by OE Biotech Co., Ltd. (Shanghai, China). Gene set enrichment analysis (GSEA) was used for pathway enrichment analysis. Molecular Signatures Database (MSigDB) of Gene ontology (GO) gene sets (C5) were used for enrichment analysis. The enrichment score (ES) in GSEA was calculated by ranking the genes from the most to least significant within the two groups (i.e., ODG/R + PIN with OGD/R), the entire ranked list was then used to assess how the proteins of each gene set were distributed across the ranked list.

### Drug affinity-responsive target stabilization assay (DARTS)

A DARTS assay was conducted as described previously [38]. Approximately 1 × 10^6^ HCMECs were lysed on ice with M-PER™ (Thermo Scientific) for 30 min. Thereafter, DMSO control or 1 μM pinacidil diluted in 1X TNC buffer (50 mmol/L Tris, 50 mmol/L NaCl, and 10 mmol/L CaCl_2_, pH 7.4) was added to the cell lysate (500 mg total protein, 5 mg/mL). The samples were mixed gently and incubated overnight at 4 ℃ for sufficient ligand–protein binding. Subsequently, the lysates were digested with pronase at a 1:500 ratio (w/w) for 30 min. Finally, the loading buffer was added to the lysates and boiled for 10 min. The resulting samples were separated by SDS-PAGE and stained with Coomassie blue. Gel bands with significant differences in abundance between the pinacidil and DMSO treatments were excised and identified via liquid chromatography–mass spectrometry (LC–MS) according to our previous study [36]. The results were searched using Maxquant 2.0.0.1 based on the UniProt database (https://www.uniprot.org/).

### Statistical analysis

The data are expressed as the mean ± SEM. Normality was assessed using the Shapiro–Wilk and Kolmogorov–Smirnov tests. Statistical analyses were performed using Student’s t-test or one-way analysis of variance (ANOVA), followed by post hoc tests. Statistical significance was set at *P* < 0.05.

## Results

### Pinacidil administration alleviates cardiac dysfunction after I/R injury in a mouse model

**Fig. 1:**
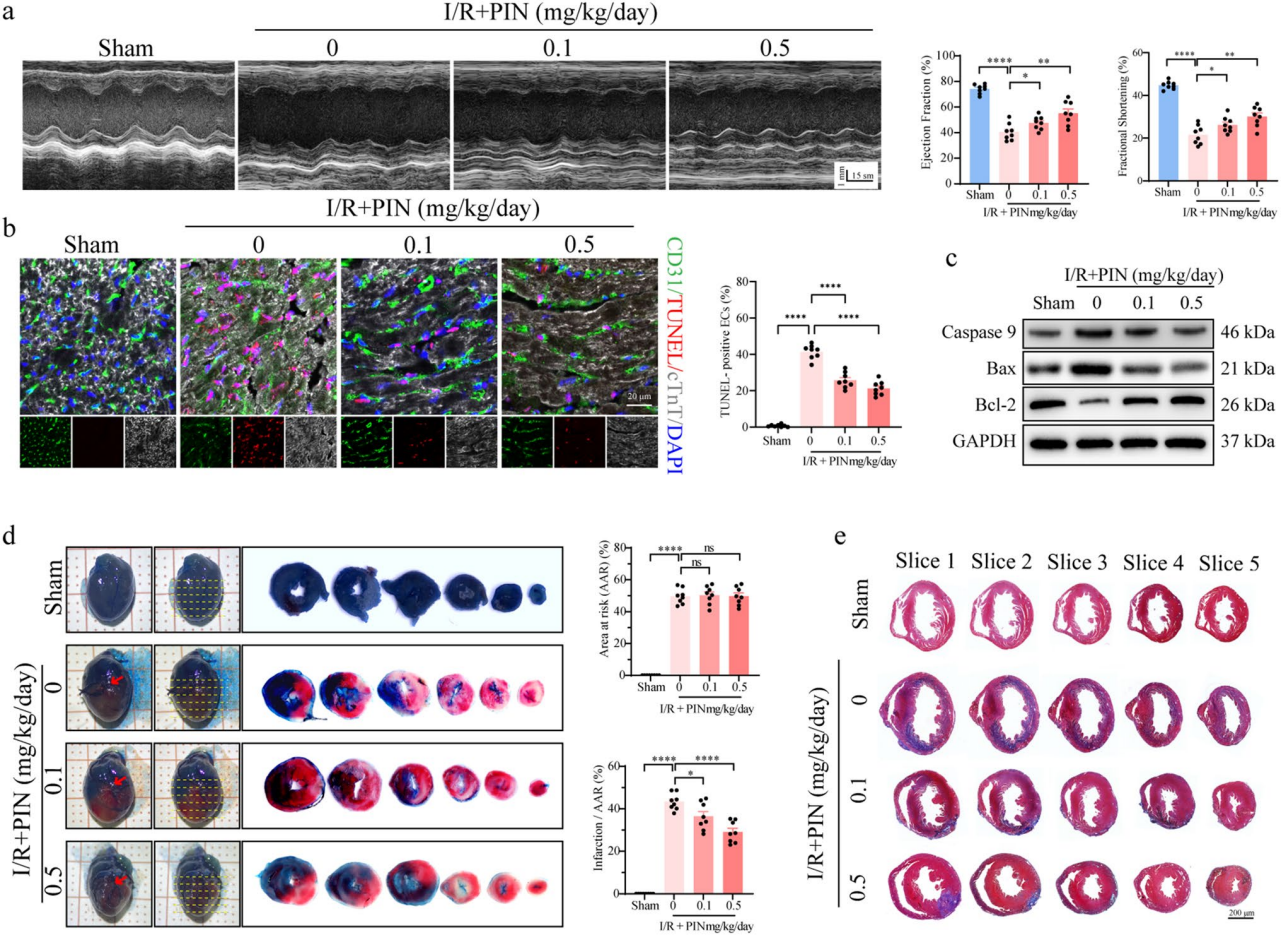
Pinacidil administration improves cardiac performance in I/R in a mouse model. **a** Cardiac functions were measured 3 days after I/R injury. Representative images of echocardiography, and statistical analysis of the LVEF and LVFS. **b** Representative images of TUNEL (red), CD31 (green), and cTnT (white, pseudo-color), and statistical analysis of TUNEL-positive endothelial cells. **c** Western blotting analysis of Bax, Bcl-2, and Caspase 9. **d** Myocardial infarction size was detected by Evans blue / TTC staining, the AAR and infarction size were quantified. **e** Representative images and statistical analysis of fibrosis scar measured by Masson’s staining. PIN: pinacidil. * *P* < 0.05, significant difference between indicated groups; ns, nonsignificant

Echocardiography was performed 3 days after I/R injury and the results showed evidently decreased left ventricular ejection fraction (LVEF) and left ventricular fractional shortening (LVFS) levels and significantly increased in the left ventricular end-diastolic diameter (LVEDD). Besides, serum B-type natriuretic peptide (BNP) levels were significantly increased. However, the above cardiac dysfunctions were improved by administration of pinacidil at 0.1 and 0.5 mg/kg/day **(**Fig. 1a, S1a-b). Additionally, systemic hemodynamics were assessed using pressure–volume loop analysis, and the results revealed that the ESPVR, + dP/dt, and -dP/dt significantly decreased after I/R injury, and EDPVR and LVEDP were significantly increased, and those hemodynamic abnormalities were alleviated by pinacidil treatment (Table S3**)**.

Apoptotic injury is a fundamental pathology of cardiac I/R injury. In the present study, the number of total TUNEL-positive cells and TUNEL-positive endothelial cells increased in the border zone of infarction (Fig. 1b). In addition, western blotting revealed that the pro-apoptotic proteins Caspase-9 and Bax were markedly upregulated, and the anti-apoptotic protein Bcl-2 was downregulated in ischemic tissues (Fig. 1c). In contrast, pinacidil treatment reduced the above-mentioned cardiac and endothelial apoptotic injury (Fig. 1b-c, S1c). Moreover, pinacidil treatment significantly reduced the infraction size after I/R injury, indicating that pinacidil improved myocyte viability and reduced myocardial necrosis (Fig. 1d). Furthermore, pinacidil administration reduced ventricular remodeling, resulting in less cardiac dilation and interstitial fibrosis (Fig. 1e, S1d).

Overall, these results suggest that pinacidil treatment alleviates I/R-induced cardiac dysfunction and remodeling, at least in part, by ameliorating cardiac apoptosis.

### Pinacidil treatment improves cardiac vascular function after I/R injury

**Fig. 2:**
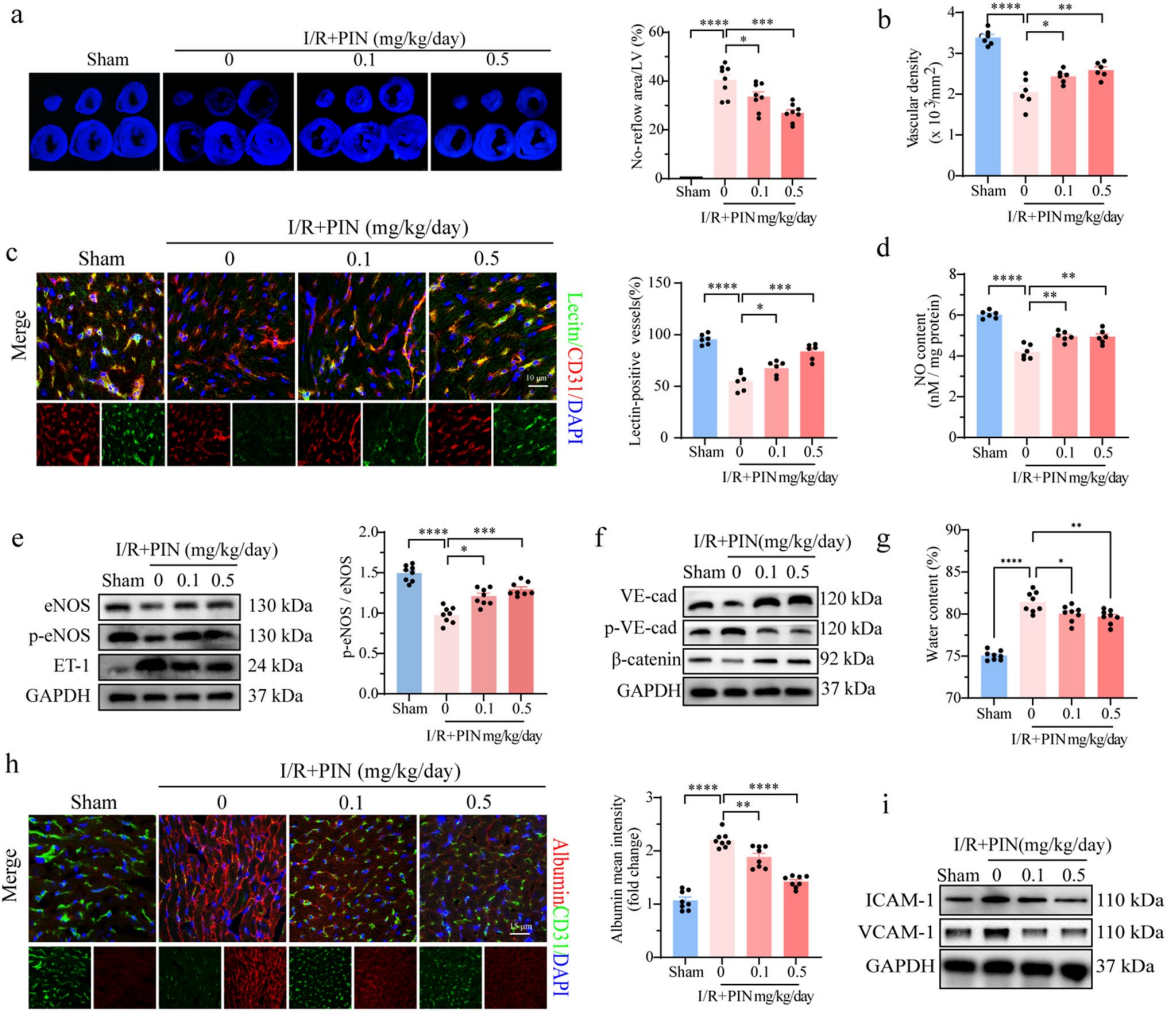
Pinacidil treatment alleviates cardiac microvascular dysfunction in I/R injury mouse model. **a** Representative images of thioflavin-S staining, and statistical analysis of the no-reflow area. **b**, **c** Representative images of CD31 staining and Lectin perfusion assay. The capillary density and perfusion were statistically analyzed. **d** NO content in the LV. **e** Endothelial eNOS phosphorylation at Ser^1177^ and ET-1 expression were detected by western blotting analysis. **f** VE-cadherin expression and phosphorylation at Tyr^731^ and β-catenin expression were detected by western blotting analysis. **g** Quantification of cardiac water content. **h** Representative immunofluorescence images and statistical analysis of albumen leakage (red). **i** Representative immunoblots VCAM-1 and ICAM-1 expression. *P < 0.05, significant difference between indicated groups; ns, nonsignificant

Vascular blood perfusion functions in cardiac repair. Therefore, we determined the specific role of pinacidil in vascular function following I/R damage. The no-reflow areas were measured using thioflavin-S staining. There was an obvious defect area visualized by Thioflavin-S staining in the LV after I/R injury, which was reduced after pinacidil treatment, indicating that pinacidil improved the vascular reperfusion and alleviated the no-reflow phenomenon (Fig. 2a). More specifically, capillary density and perfusion significantly decreased after I/R injury, which was also improved by pinacidil administration (Fig. 2b-c). Whereas the blood perfusion of arterioles was not compromised by I/R injury (Fig. S1e). In addition, we detected a significant decrease in NO generation, upregulation of endothelin-1 (ET-1) expression, downregulation of endothelial NO synthase (eNOS) expression, and phosphorylation after I/R injury. (Fig. 2d-e). In contrast, these effects were alleviated by pinacidil treatment in a dose-dependent manner, indicating a potential role of pinacidil in improving vascular perfusion via endothelium-dependent vasodilation (Fig. 2d-e).

Endothelial barrier collapse is another feature of I/R injury that leads to long-term cardiac edema and myocardial fibrosis. Western blotting revealed that vascular endothelial (VE)-cadherin and β-catenin expression decreased and the phosphorylation of VE-cadherin at Tyr^731^ increased after I/R injury, indicating the weakening of the endothelial anchoring connection (Fig. 2f). Moreover, increased albumin leakage and water content were detected after I/R injury (Fig. 2g, h). However, these endothelial barrier dysfunctions were overtly improved after pinacidil treatment, suggesting that pinacidil may alleviate vasogenic edema (Fig. 2g, h). Intercellular adhesion molecule-1 (ICAM-1) and vascular adhesion molecule-1 (VCAM-1) are two vascular molecules that facilitate leukocyte adhesion and exudation. After cardiac I/R injury, the expression levels of ICAM-1 and VCAM-1 increased, indicating the activation of endothelium-dependent inflammation (Fig. 2i). Importantly, pinacidil treatment largely reversed the upregulation of ICAM-1 and VCAM-1 expression, indicating its potential role in alleviating vascular inflammatory injury (Fig. 2i).

These results suggest that pinacidil treatment protected microvascular function against cardiac I/R injury by improving vascular perfusion, enhancing endothelial barrier function, and inhibiting endothelium-dependent inflammation.

### Pinacidil maintains HCMEC function after OGD/R injury by relieving mitochondria-dependent apoptosis

**Fig. 3:**
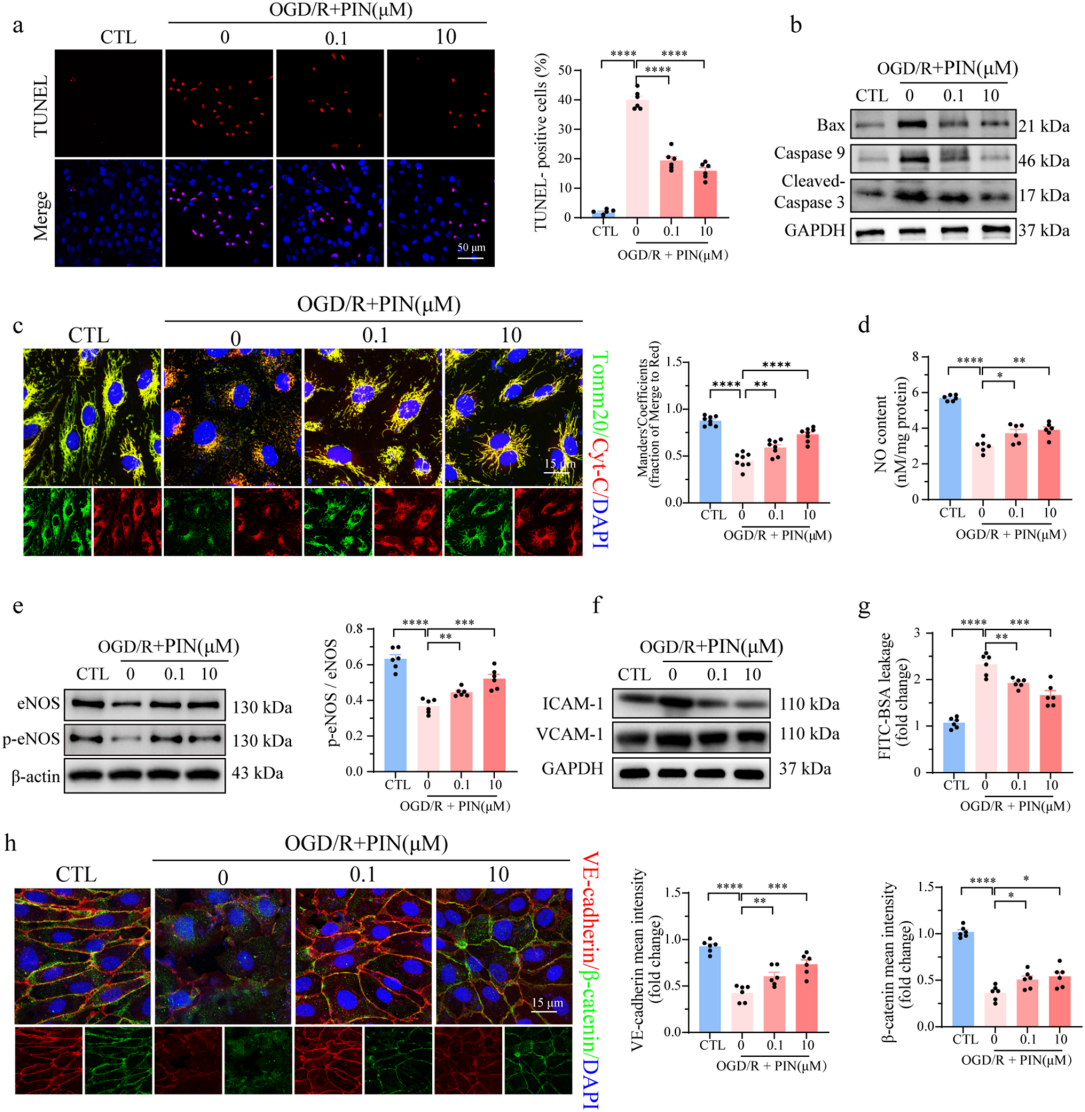
Pinacidil improves HCMECs functions and survival in OGD/R injury. **a** Representative images and quantitative analysis of the TUNEL assay. **b** Representative western blotting of Bax, Caspase 9, and cleaved-Caspase3 expression. **c** Representative immunofluorescence images and quantification of mitochondrial Cyt-C release (red). **d** NO content in the culture medium. **e** eNOS expression and phosphorylation at Ser^1177^ were detected by western blotting analysis. **f** Western blotting analysis of the protein expression of ICAM-1 and VCAM-1. **g** Endothelial monolayer permeability was evaluated by FITC–BSA leakage assay. **h** Representative images and quantitative analysis of VE-cadherin (red) and β-catenin (green). *P < 0.05, significant difference between indicated groups; ns, nonsignificant

To elucidate the potential mechanism of pinacidil in endothelial protection, we assessed the viability, proliferation, and apoptosis of HCMECs after OGD/R injury. OGD/R injury significantly reduced cell viability and proliferation, which were ameliorated by pinacidil treatment in a dose-dependent manner (Fig. S1f-g). Consistent with its protective effect against endothelial apoptosis in the I/R hearts, pinacidil also reduced the ratio of TUNEL-positive HCMECs following OGD/R injury (Fig. 3a). Meanwhile, the mitochondria-related apoptosis proteins Bax, Caspase 9, and cleaved-Caspase 3 were upregulated in OGD/R injury but were downregulated after pinacidil treatment (Fig. 3b). In addition, the negative effects of cytochrome C (Cyt-C) release into the cytoplasm following OGD/R injury were remarkably decreased by pinacidil treatment. Collectively, the above results indicated that pinacidil protects HCMECs against OGD/R injury by inhibiting mitochondria-dependent apoptosis pathways (Fig. 3c).

Further, we investigated the effect of pinacidil on endothelial function. Following OGD/R injury, a significant decrease in the NO content and eNOS expression and its Ser^1177^ phosphorylation was observed, which was largely restored by pinacidil treatment (Fig. 3d-e). In addition, the expression levels of ICAM-1 and VCAM-1 were rapidly increased in OGD/R injury. However, this endothelial inflammatory response was evidently alleviated by pinacidil treatment (Fig. 3f). Additionally, pinacidil treatment reduced FITC–BSA leakage in OGD/R injury, indicating a role of the drug in the protection of endothelial barrier function (Fig. 3g). Immunofluorescence analysis showed that OGD/R injury resulted in the morphological destruction of β-catenin and VE-cadherin, visualized by their reduced fluorescence intensities. However, pinacidil treatment simultaneously improved the intensity and morphology of β-catenin and VE-cadherin to a large extent, indicating the ability of pinacidil to repair endothelial barier junctions (Fig. 3h).

Taken together, the above evidence confirms that pinacidil has beneficial effects on endothelial protection, at least in part, by suppressing mitochondria-dependent apoptosis.

### Pinacidil relieves calcium overload after OGD/R injury in HCMECs

**Fig. 4:**
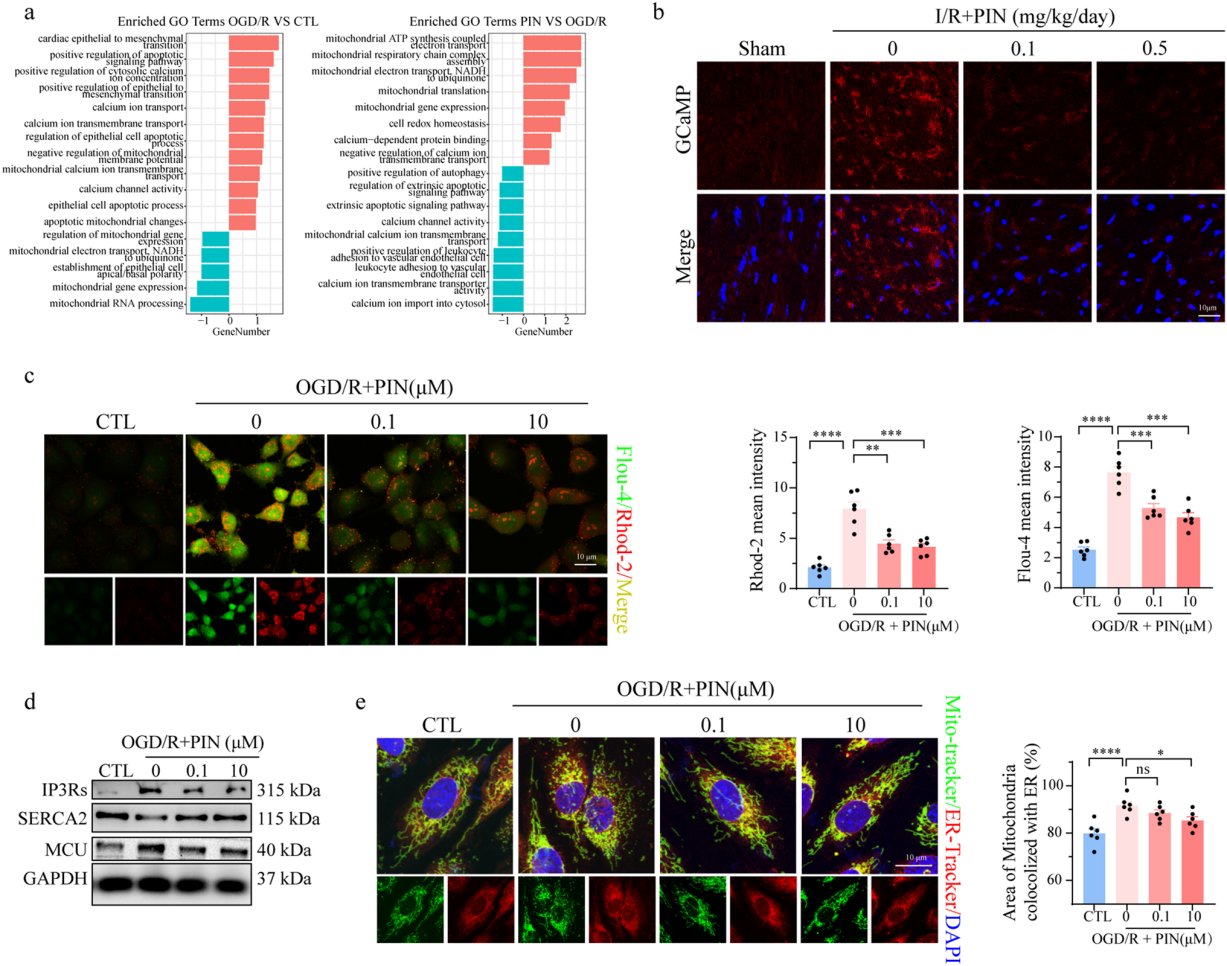
Pinacidil treatment reduces calcium overload after OGD/R injury. **a** RNA-sequencing assay among the control (CTL), OGD/R, and OGD/R + pinacidil groups. Enriched pathways of DEGs demonstrated by GO biological processes. **b** Representative images of real-time calcium reporter GCaMP8 in cardiac microcirculation in the indicated groups. **c** Representative images and quantitative analysis of [Ca^2+^]_i_ (green) and [Ca^2+^]_m_ (red) in OGD/R-injured HCMECs. **d** Expression of IP3Rs, SERCA2, and MCU in HCMECs were detected by western blotting analysis. **e** Representative fluorescence image and quantitative analysis of colocalization between mitochondria and ER. *P < 0.05, significant difference between indicated groups; ns, nonsignificant

To further elucidate the potential mechanisms underlying pinacidil’s protective effect on endothelial cells, we performed mRNA sequencing assays on OGD/R-injured HCMECs, with or without pinacidil treatment. GO term enrichment revealed significant enrichment of differentially expressed genes (DEGs) related to mitochondrial function, ER stress pathways, and calcium related pathways (Fig. 4a, Fig. S2a-b), In addition, the GSEA results revealed significant enriched pathways related to mitochondrial respiratory chain complex assembly, calcium transmembrane transporter activity, and calcium-dependent protein binding after pinacidil treatment, further supporting the potential benefits of pinacidil on mitochondrial function and calcium signaling (Fig. S2c), while there were seldom DEGs and enriched pathways in the two normoxia groups.

To further verify the above assumption, we recorded the calcium overload via real-time calcium signal reporter GCaMP8 in endothelium. After I/R injury, the red calcium fluorescence was significantly higher than that of sham mice, indicating endothelial calcium overload was activated after I/R injury. In contrast, pinacidil significantly reduced the fluorescence intensity in I/R hearts. (Fig. 4b). Further, the benefits of pinacidil on mitochondrial function and intracellular calcium fluctuations were validated in vitro. Following OGD/R injury, the mitochondrial networks in HCMECs became disorganized and fragmented, but were repaired by pinacidil treatment (Fig. S2d). Meanwhile, mitochondrial membrane potential was significantly decreased after OGD/R injury, and was improved in pinacidil treatment (Fig. S2e). Moreover, OGD/R injury resulted in a cellular ROS burst, together with mtROS accumulation, whereas pinacidil treatment suppressed both the cellular ROS burst and mtROS accumulation (Fig. S2f). More importantly, OGD/R injury significantly upregulated the [Ca^2+^]_i_ and [Ca^2+^]_m_ levels, which were largely reversed by pinacidil treatment (Fig. 4c).

Calcium homeostasis is primarily regulated by member calcium channels and shuttles between the ER and mitochondria. In the present study, we found significantly increased MCU and IP3Rs levels, along with a significant reduction in SERCA2 levels, in OGD/R-injured HCMECs, which was reversed by pinacidil treatment (Fig. 4d). Additionally, the overlapping regions of the ER and mitochondria, known as MAMs, were markedly decreased by pinacidil treatment (Fig. 4e). These results strongly suggest that the protective effects of pinacidil against mitochondrial injury largely depend on reducing calcium shuttling from the ER to mitochondria, likely through the modulation of MCU–IP3Rs–SERCA2 pathways and MAMs formation.

### Pinacidil reduces chaperone-mediated autophagy (CMA) of CRT

**Fig. 5:**
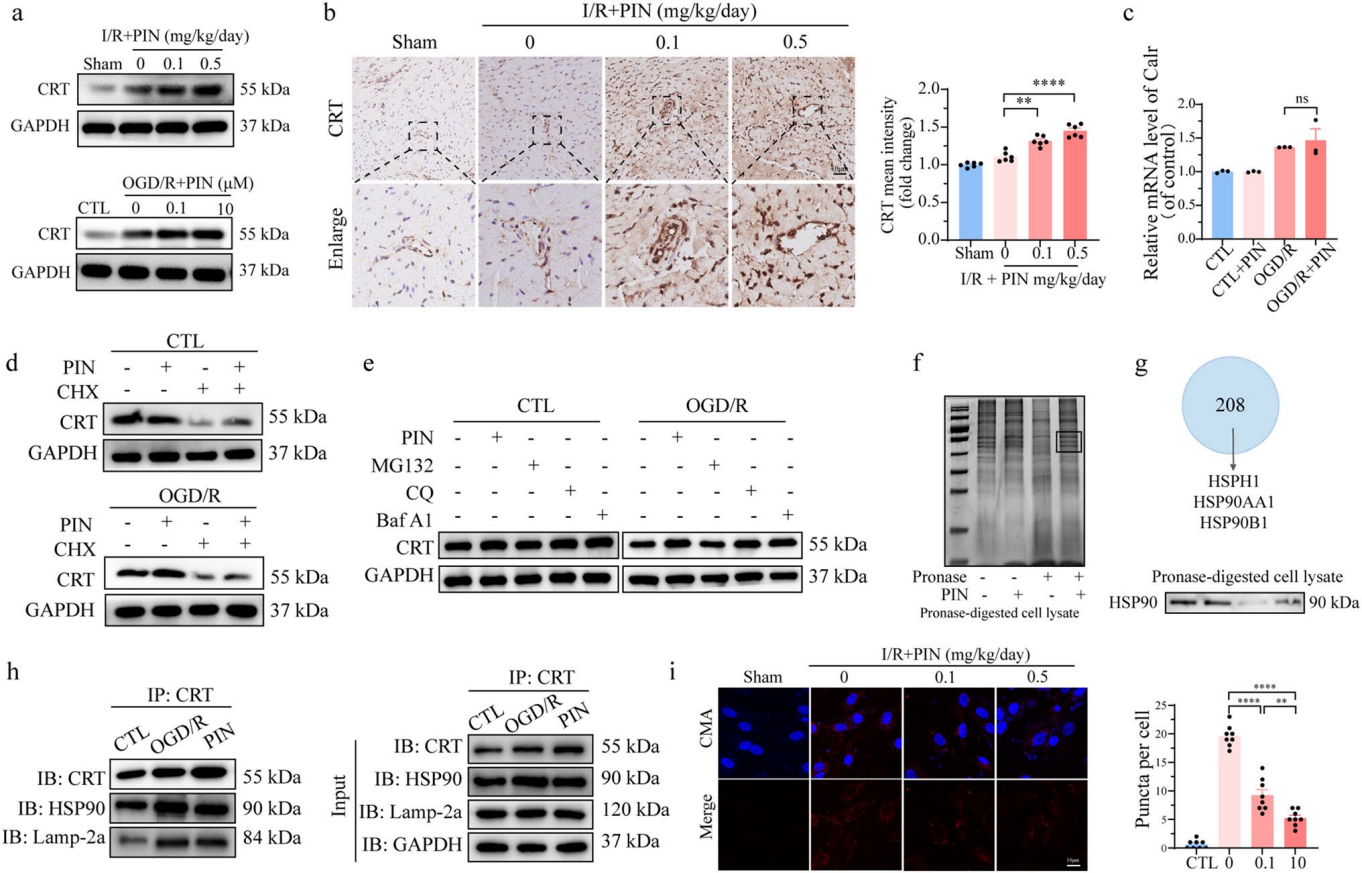
Pinacidil inhibits CMA of CRT in OGD/R injury. **a** CRT protein expression in mouse models and HCMECs were detected by western blotting analysis. Quantitative analysis of CRT protein expression in mice. **b** Representative immunohistochemistry images and quantitative analysis of the CRT expression. **c** Relative mRNA expression of CRT. **d** Representative immunoblots of the CRT expression after CHX treatment. **e** Western blotting analysis of CRT expression in HCMECs pretreated with MG132, CQ, and Baf A1. **f** A marked increase in band intensity near 90 kDa in pronase-digested cell lysates were detected by DARTS assay after pinacidil treatment. **g** LC–MS was conducted on the 90-kDa gel (black frame). Western blotting analysis of HSP90 expression in pronase-digested cell lysates. **h** Co-IP assays were performed using CRT antibody and then western blotting was performed for CRT, HSP90, and Lamp-2a. **i** Representative fluorescent images of mCherry-KFERQ-NE in primary MCMECs isolated from I/R mice, with or without pinacidil treatment. *P < 0.05, significant difference between indicated groups

Based on the pinacidil’s contribution to calcium homeostasis, especially in the ER–mitochondrial calcium shuttle, experiments were performed to further explore the underlying molecular mechanisms. CRT is a calcium buffer located in the ER and maintains the intercellular calcium homeostasis. In the present study, we found that OGD/R injury upregulated CRT expression (Fig. 5a). Interestingly, pinacidil administration promoted CRT protein expression in a dose-dependent manner (Fig. 5a, S5a) and increased CRT expression in mouse I/R hearts, especially in the vascular endothelium (Fig. 5b).

Further experiments were conducted to determine how pinacidil regulates CRT protein expression. After OGD/R injury, the mRNA expression was increased, which could partly explain the increased protein expression (Fig. 5c). However, pinacidil did not increase CRT mRNA expression after OGD/R injury (Fig. 5c). Hence, we hypothesized that pinacidil may regulate CRT protein stability and degradation. After inhibiting protein transcription via cycloheximide (CHX) treatment, addition of pinacidil maintained CRT protein expression under both normal and OGD/R conditions, suggesting that pinacidil inhibited CRT protein degradation. (Fig. 5d). We then evaluated the change in CRT protein levels with proteasome and lysosome inhibitors and found that only the lysosome inhibitors CQ and Baf A1 could increase CRT protein levels after OGD/R injury (Fig. 5e). Moreover, OGD/R injury increased lysosomal CRT levels, which was inhibited by pinacidil treatment (Fig. S3b). These results confirm that pinacidil may reduce the autophagic degradation of CRT after OGD/R injury.

To explore the exact mechanism by which pinacidil regulates the autophagic degradation of CRT, a DARTS assay was conducted to identify proteins that directly interact with pinacidil. The assay, identified a group of proteins shown by an intense gel band above 90 kDa after pinacidil treatment (Fig. 5f). The LC–MS analysis identified HSP family members, especially HSP90B1, HSP90AA1, and HSPH1, as the top proteins in this specific gel band (Fig. 5g). Western blotting also revealed that pinacidil protected HSP90 against pronase, further confirming the potential of pinacidil to directly bind to HSP90 (Fig. 5g). Based on its involvement in CMA of protein degradation, we investigated whether HSP90 plays a role in modulating pinacidil-induced CRT protein expression. IP assays suggested a mutual interaction between HSP90 and CRT (Fig. S3c). In addition, pinacidil treatment significantly reduced the interaction between CRT and HSP90 in both lysosomes and the cytoplasm (Fig. 5h), that pinacidil maintains CRT expression mainly by inhibiting the HSP90-dependent CMA of CRT. Most importantly, we measure the CMA in primary MCMECs form I/R-injured mice, with or without pinacidil treatment, as described previously [26]. I/R injury remarkably increased the CMA puncta number in endothelial cells, an effect was reduced by pinacidil treatment (Fig. 5i). Therefore, the inhibition of HSP90-mediated CMA degradation of CRT may be one of the potential molecular mechanisms underlying the benefits of pinacidil on endothelial calcium homeostasis.

### Overexpression of CRT relives calcium overload and rescues endothelial cell functions after OGD/R injury

**Fig. 6:**
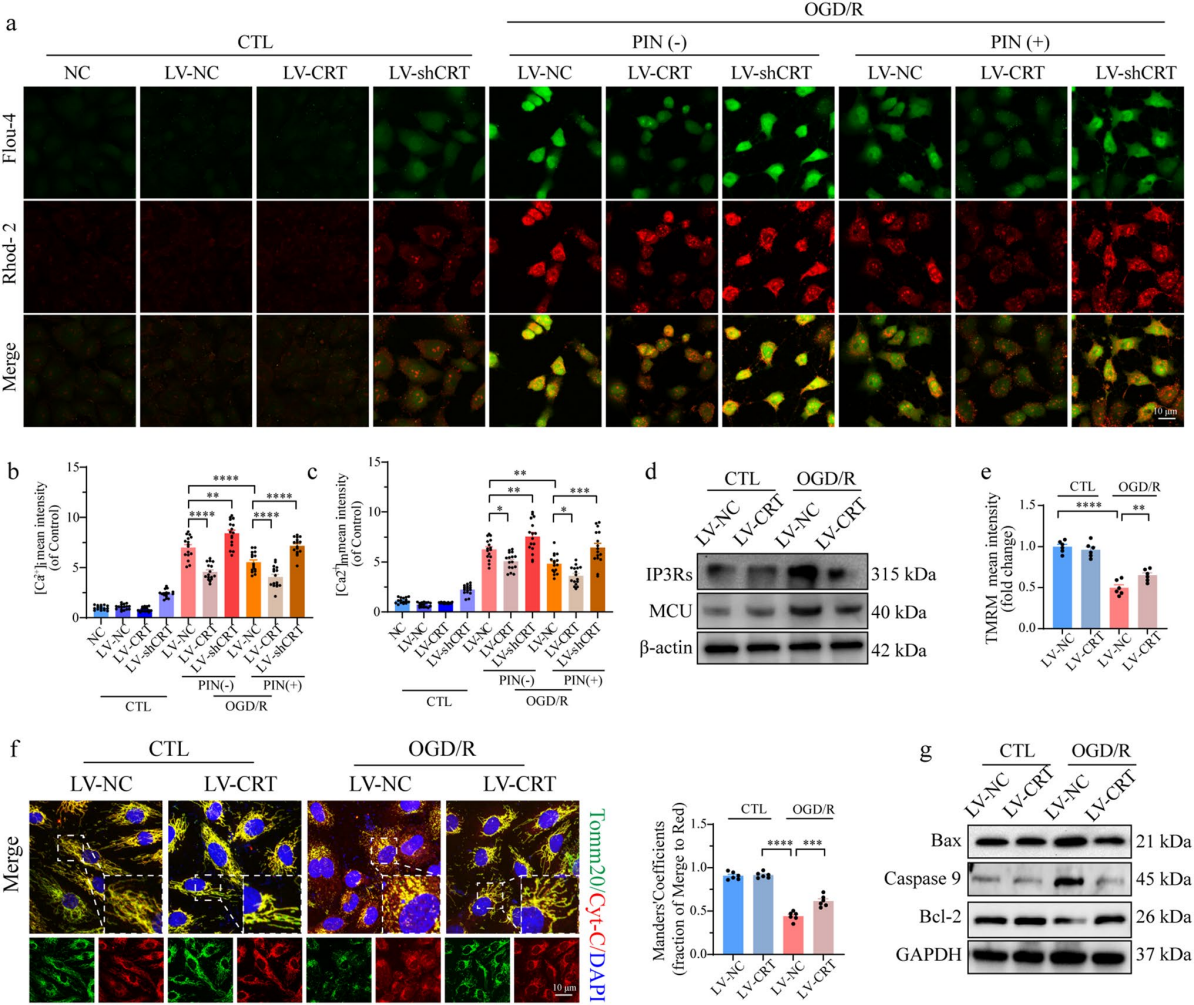
CRT overexpression alleviates calcium overload and mitochondrial dysfunction in OGD/R-injured HCMECs. **a**, **c** [Ca^2+^]_i_ and [Ca^2+^]_m_ content was determined by Flou-4 and Rhod-2 fluorescence staining, respectively, and quantitatively analyzed. **d** Expression of IP3Rs and MCU were analyzed by western blotting. **e** Representative immunofluorescence images and quantitative analysis of the TMRM intensity. **f** Representative immunofluorescence images and quantification of mitochondrial Cyt-C release (red). **g** Bax, Caspase 9, and Bcl-2 expression were detected by western blotting analysis. *P < 0.05, significant difference between indicated groups; ns, nonsignificant

Further attempts were made to verify the benefits of CRT on endothelial protection (Fig. S4a). Under normal conditions, CRT overexpression had no effect on calcium overload **(**Fig. 6a-c**)**. However, after OGD/R injury, CRT overexpression protected HCMECs from mitochondrial and cellular calcium overload, showing the same results as pinacidil treatment (Fig. 6a-c). Similarly, the calcium tunnels IP3Rs and MCU were downregulated by CRT overexpression after the reoxygenation injury, indicating that less calcium was transferred from the ER to mitochondria (Fig. 6d). Along with improved mitochondrial membrane potential, the above results indicated that mitochondrial dysfunction was ameliorated by CRT overexpression (Fig. 6e). In addition, CRT overexpression reduced intracellular and mitochondrial ROS levels after OGD/R injury (Fig. S4c). Following improvement in mitochondrial function, mitochondria-related apoptosis was detected. CRT overexpression restricted Cyt-c release into the cytoplasm, downregulated Bax and Caspase-9 expression, and upregulated Bcl-2 expression after OGD/R injury, strongly indicating the inhibitory role of CRT in mitochondria-related apoptosis (Fig. 6f-g).

Subsequently, we determined whether the inhibitory effects of CRT on calcium homeostasis protected endothelial function. CRT overexpression significantly restored NO generation and eNOS expression and phosphorylation following OGD/R injury, indicating rescue of endothelium-dependent vascular relaxation (Fig. S5a-b). Moreover, CRT overexpression alleviated endothelial-involved inflammation, as evidenced by reduced ICAM-1 and VCAM-1 expression levels (Fig. S5c). After OGD/R injury, the endothelial barrier function was compromised, as indicated by increased FITC–BSA leakage, reduced VE-cadherin and β-catenin levels, and increased Tyr^731^ phosphorylation of VE-cadherin. However, these injuries were significantly alleviated by CRT overexpression (Fig. S5d-e). Furthermore, we demonstrated that the protection provided by CRT facilitated cell migration and angiogenesis, as indicated by faster wound healing and more angiogenic branch points in the treated group compared to the untreated group (Fig. S5f-g).

In summary, the present data showed a positive effect of CRT on endothelial protection against OGD/R injury via its role in maintaining calcium homeostasis, improving mitochondrial function, and inhibiting mitochondrial apoptosis.

### CRT knockdown abolishes the protective effect of pinacidil on mitochondrial and endothelial functions

Further investigation was conducted to elucidate the role of CRT in the therapeutic effect of pinacidil. The present data showed that CRT knockdown led to more severe calcium overload and higher expression of IP3Rs and MCU in OGD/R-injured HCMECs compared to control cells (Fig. 6a-c, S6a). In addition, the beneficial effects of pinacidil on calcium homeostasis were significantly abolished after CRT knockdown (Fig. 6a-c, S6a). In addition, CRT ablation augmented mitochondrial dysfunctions and accentuated mitochondria-dependent apoptosis under OGD/R injury, as evidenced by decreased TMRM intensity, upregulated Bax and Caspase 9 expression, downregulated Bcl2 expression, and increased number of TUNEL-positive cells (Fig. S6b-d). Similarly, the therapeutic effects of pinacidil on improving mitochondrial dysfunction and mitochondrial-dependent apoptosis were abolished after CRT knockdown (Fig. S6b-d).

More importantly, CRT knockdown exacerbated endothelial dysfunction. It further reduced NO generation after OGD/R injury and abolished the protective effects of pinacidil treatment (Fig. S6e). In addition, CRT knockdown enhanced endothelial barrier collapse by reducing the expression of VE-cadherin and β-catenin, increased FITC–BSA leakage and enhanced the endothelial inflammatory response by upregulating VCAM-1 and ICAM-1 (Fig. S6f-g). It is reasonable to predict that CRT ablation would similarly abrogate the protective effects of pinacidil on the endothelial barrier function and inflammatory response (Fig. S6f-g).

These findings indicate that CRT deficiency accelerates calcium overload and abolishes the beneficial effects of pinacidil in repairing mitochondrial function and inhibiting mitochondria-dependent apoptosis, leading to the deterioration of endothelial function after OGD/R injury.

### Overexpression of CRT alleviates cardiac microvascular I/R injury in a mouse model

**Fig. 7:**
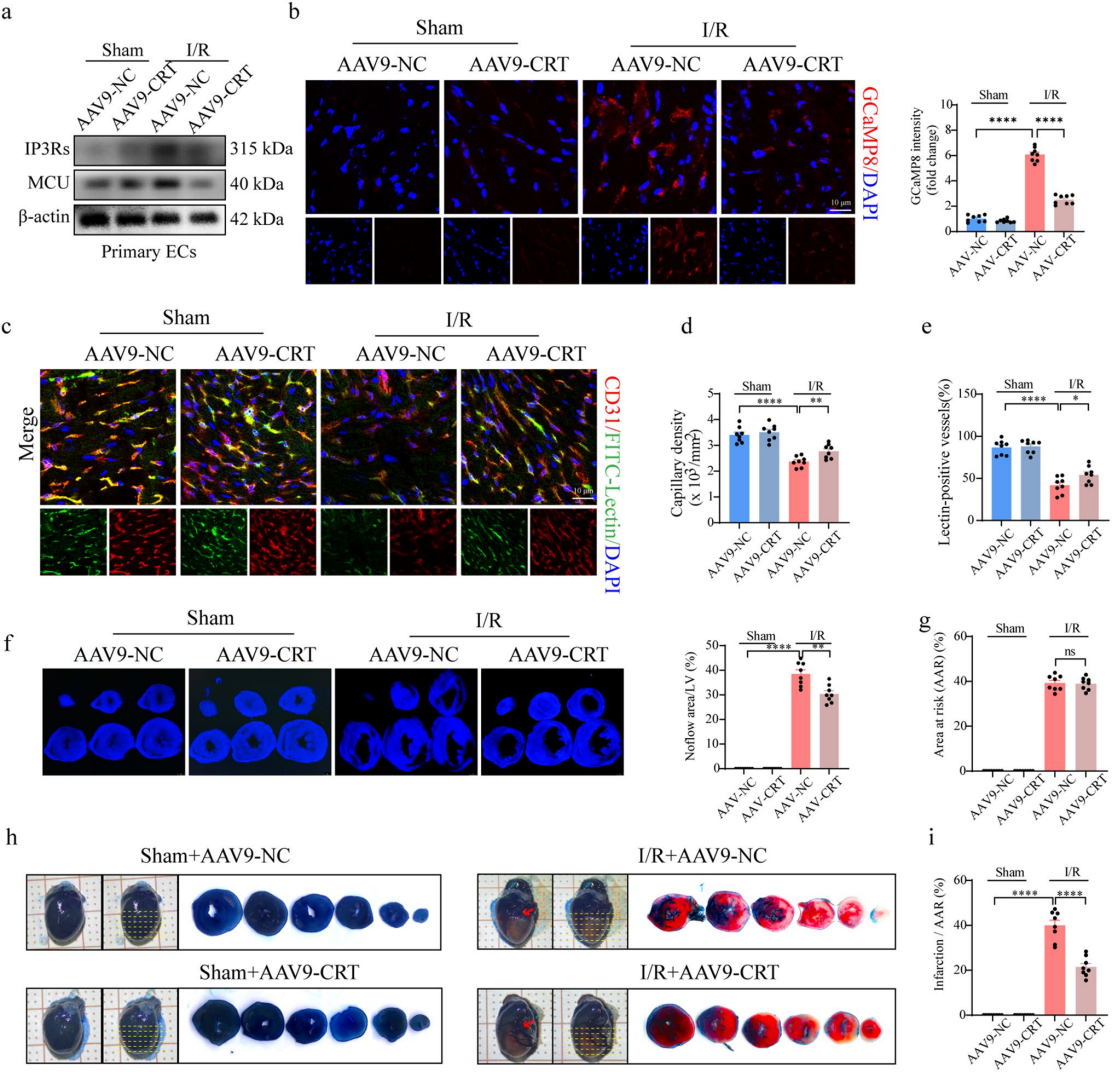
Endothelial-specific CRT overexpression alleviates cardiac microcirculation injury and reduced infarction size in I/R injury. **a** Expression of IP3Rs and MCU in primary MCMECs were detected by western blotting analysis. **b** Representative images and quantitative analysis of GCaMP8 intensity. **c**–**e** Representative images of CD31 staining and lectin perfusion assay. The capillary density and perfusion were statistically analyzed. **f** No-reflow area was detected by thioflavin-S staining and quantified. **g**, **h**, **i** Infarct size was detected by Evans blue/TTC staining and quantified. *P < 0.05, significant difference between indicated groups; ns, nonsignificant

To further validate the role of CRT in endothelial calcium homeostasis and cardiac microvascular protection, we established an AAV9-mediated endothelium-specific CRT-overexpressing mouse model. The fluorescence intensity of CRT was significantly higher in the Flag-positive areas of AAV9-CRT-infected hearts than those of AAV9-NC-infected hearts (Fig. S7a). After CRT overexpression, IP3Rs and MCU expression was significantly suppressed in primary HCMECs (Fig. 7a). Additionally, CRT overexpression significantly decreased the fluorescence intensity of GCaMP8 in I/R-injured mice, indicating its potential to reduce endothelial calcium overload (Fig. 7b). In addition, endothelial-specific overexpression of CRT significantly decreased the endothelial cell apoptosis, enhanced capillary density and perfusion, and alleviated the no-reflow phenomenon after I/R injury (Fig. 7c–f, S7b-e). These data confirm the beneficial effects of CRT on endothelial calcium homeostasis, endothelial survival, and vascular perfusion.

Given these findings, we sought to determine whether the protective effects of CRT overexpression on microcirculation benefited the cardiac IR injury. Similar to pinacidil treatment, endothelial-specific overexpression of CRT significantly reduced the infarction size and improved cardiac function (Fig. 7g–i, Fig, S7f). Additionally, Pressure–volume loop analyses indicated that CRT overexpression improved the ESPVR and EDPVR in I/R injury, consistent with the aforementioned echocardiography results (Table S4). The above data collectively demonstrated that CRT overexpression-induced vascular improvement benefits the cardiac functions.

Combine with our previous findings, we conclude that pinacidil protects the cardiac microcirculation and endothelial cells against reperfusion/reoxygenation injury via CRT-mediated calcium homeostasis and mitochondrial protection.

## Discussion

The major findings of the present study demonstrated the beneficial effects of pinacidil on endothelial and cardiovascular protection following cardiac I/R injury. Pinacidil treatment inhibited the CMA degradation of CRT by directly binding to HSP90. Importantly, upregulated CRT expression inhibited calcium overload-induced mitochondrial dysfunction and mitochondria-dependent apoptosis via the IP3Rs/MCU signaling pathway, ultimately improving endothelial function and cardiac microvascular I/R injury (Scheme 1).

Cardiac microvascular dysfunction occurs in 5–50% of myocardial infarction patients even after successful PCI [13]. An imbalance between endothelial vasodilating and vasoconstricting factors can lead to a no-flow or low-reflow area phenomenon, further exacerbating cardiac infarction and dysfunction. Therefore, it is imperative to improve the cardiac microcirculation [28]. K_ATP_ channels are important regulators of coronary microcirculation via dilating arterioles via inhibition of the calcium influx in VSMCs [27]. Besides, endothelial K_ATP_ paly synergistical role in cardiovascular protection. Endothelial K_ATP_ channels regulate the alterations of coronary microcirculation flow in response to metabolic demand [42]. Moreover, endothelial K_ATP_ channels assists VSMCs contraction by directly affecting the VSMCs membrane potential and indirectly releasing of vasoactive substances [1, 10]. Interestingly, there is strong evidence that the coronary vasodilation is majorly mediated by endothelium-dependent relaxation, considering the activation of VSMCs K_ATP_ channels relies on endothelium-derived factors and the removal of endothelium decreases the sensitivity of arteries to K_ATP_ channel openers [22, 37]. The present study used pinacidil, a nonselective opener of K_ATP_ channels, to investigate endothelial K_ATP_ channels in cardiovascular protection and demonstrated that pinacidil alleviated the endothelial calcium overload, reduced mitochondria-related apoptosis, improved the capillary density, and therefore enhanced blood perfusion and reduced the no-reflow area, which indicated a significant role of endothelial K_ATP_ channel in cardiac microvascular I/R injury.

NO is important regulators of coronary circulation, and NO dilates mainly larger arterioles [27, 52]. A previous study revealed that administration of NO, NO donors, or drugs that enhance NO release protect the myocardium against I/R injury [49]. On the other hand, another study concluded that the K_ATP_ channel activation, but not NO, is a major mechanism of protection against microvascular injury, since there were no statistically significant differences in infarct size and no-reflow zone in NO activator-treated I/R rabbits [15]. In the present study, pinacidil enhanced the expression and phosphorylation of eNOS, increased NO content and synthesis, which further improved endothelial dysfunction and attenuated infarct size, indicating that pinacidil may improve cardiac microvascular injury via both K_ATP_ opening and NO-dependent vasodilatation.

Endothelial calcium overload is considered the core mechanism underlying cardiac microvascular damage after I/R injury [33, 46]. The impairment of calcium homeostasis in endothelial cells modulated the connexins, gap junction, and endothelium permeability, finally increased the water permeability and protein leakage [14]. On the other hand, abnormality of intracellular calcium overload increased intracellular osmotic gradient, induced the water influx and promoted cardiomyocytes swelling and intracellular edema [2]. The present study found pinacidil treatment reduced the vascular leakage via enhancing endothelial anchoring junctions (VE-cadherin and β-catenin), suggesting a crucial role of K_ATP_ and calcium hemostasis in the maintain of endothelial barrier function.

With respect to calcium regulatory machinery in the ER, upregulation of IP3Rs and downregulation of SERCA2 have been noted in ischemic hearts and are responsible for mitochondrial calcium overload by transferring calcium from the ER to mitochondria [67]. Earlier studies have shown that inhibition of IP3Rs or overexpression of SERCA2 significantly reduced endothelial mitochondrial calcium overload and protected against I/R-induced microcirculation dysfunction [6, 33]. The MCU is another calcium channel that is specifically located in the mitochondria and receives calcium released from IP3Rs in MAMs [47, 59]. Our previous study revealed that the activation of the MCU complex in endothelial cells induces mitochondrial calcium influx, mitochondrial fission, mitochondrial Cyt-C release, and mitochondria-dependent apoptosis, ultimately leading to cardiac microvascular I/R injury [34].The present work further confirmed that pinacidil and CRT overexpression reduced IP3Rs and MCU expression, increased SERCA2 expression, and suppressed MAMs formation,, ultimately reduced endothelial calcium overload.

As a highly selective form of autophagy, CMA degrades proteins with molecular chaperones, including HSP70 and HSP90, which transport target proteins to the lysosomes [23, 60]. Dysregulated CMA has been implicated in multiple cardiovascular diseases, such as atherosclerosis, doxorubicin cardiomyopathy and heart failure [3]. In the present study, pinacidil pharmacologically inhibited the CMA degradation of CRT, thereby increasing its stability and expression. CRT has been indicated to interact with HSP90 [63]. In this study, we demonstrated that pinacidil directly binds to HSP90 and blocks its interaction with CRT, resulting in less CMA degradation of CRT. The above results suggested that pinacidil exerted a protective effect on endothelial cells by reducing HSP90-mediated CMA degradation of CRT, thereby maintaining endothelial calcium homeostasis in reperfusion injury.

In addition to the well-known benefits of pinacidil in improving hypertension by relieving vasoconstriction, our study further verifies its benefits on endothelial protection by reducing the CMA degradation of CRT in I/R injury. However, this study has several experimental limitations. The current data used the FITC–lectin perfusion method to detect vascular blood flow, but more direct method using laser Doppler flowmetry would be more accurate [54]. The present work found pinacidil alleviated cardiac edema after I/R injury, however, discrimination between intra and extracellular myocardial edema is presently difficult at the bench and impossible at the bedside [14]. Since both the overexpression and knockdown of CRT in cardiomyocytes could cause cardiac dilation and dysfunction, further investigations are needed to elucidate the underlying mechanisms that separate from its role in endothelial protection [31, 41].

In conclusion, our study shows that both pinacidil treatment and CRT overexpression have protective effects against cardiac microvascular I/R injury. Pinacidil inhibited the CMA degradation of CRT, thereby maintaining mitochondrial calcium homeostasis and ultimately protecting the cardiac microvascular structure and function against I/R injury. These findings reveal a new mechanism for pinacidil in cardiovascular protection and suggest that the CRT/calcium overload/mitochondrial apoptosis signaling pathway is a potential pathological mechanism underlying endothelial injury.

### Supplementary Information

Below is the link to the electronic supplementary material.

Supplementary file1 (DOCX 18 KB)

Supplementary file2 (DOCX 19 KB)

Supplementary file3 (DOCX 17 KB)

Supplementary file4 (DOCX 17 KB)

Supplementary file5 (TIF 6018 KB)

Supplementary file6 (TIF 20738 KB)

Supplementary file7 (TIF 5523 KB)

Supplementary file8 (TIF 5380 KB)

Supplementary file9 (TIF 11521 KB)

Supplementary file10 (TIF 8132 KB)

Supplementary file10 (TIF 9773 KB)
