# Supplementary material for: Pinacidil ameliorates cardiac microvascular ischemia–reperfusion injury by inhibiting chaperone-mediated autophagy of calreticulin
Source: Basic Res Cardiol. 2024 Jan 2;119(1):113–31. doi: 10.1007/s00395-023-01028-8 (PMC10837255; doi:10.1007/s00395-023-01028-8)
Supplement: Supplementary file 1 — Supplementary file1 (DOCX 18 KB) [file 395_2023_1028_MOESM1_ESM.docx]

Table S1. Primary and secondary antibodies used in immunofluorescence.

| Name | Manufacturer | Cat No. | Dilution | Application |
| --- | --- | --- | --- | --- |
| cTnT | Protein Tech | 15513-1-AP | 1:500 | IHC-Fr |
| Albumen | Abcam | ab207327 | 1:200 | IHC-Fr |
| CD31 | Abcam | ab7388 | 1:500 | IHC-Fr |
| α-SMA | Abcam | Ab124964 | 1:200 | IHC-Fr |
| VE-Cadherin | Thermo Fisher | 14-1449-82 | 1:200 | ICC/IF |
| β-catenine | Abcam | Ab32572 | 1:200 | ICC/IF |
| Cytochrome C | Abcam | ab110325 | 1:200 | ICC/IF |
| Tomm20 | Abcam | ab186735 | 1:200 | ICC/IF |
| Albumin | Abcam | ab207327 | 1:200 | IHC-Fr |
| CRT | Abcam | ab92516 | 1:200 | IF |
| Donkey-anti mouse AF568 | Abcam | ab175700 | 1:500 | IHC-Fr ICC/IF |
| Donkey-anti rat AF488 | Thermo Fisher | A21208 | 1:500 | IHC-Fr ICC/IF |
| Donkey-anti rabbit AF488 | Thermo Fisher | A21206 | 1:500 | IHC-Fr ICC/IF |
| Donkey-anti rabbit AF594 | Thermo Fisher | A21207 | 1:500 | IHC-Fr ICC/IF |
