# Supplementary material for: Pinacidil ameliorates cardiac microvascular ischemia–reperfusion injury by inhibiting chaperone-mediated autophagy of calreticulin
Source: Basic Res Cardiol. 2024 Jan 2;119(1):113–31. doi: 10.1007/s00395-023-01028-8 (PMC10837255; doi:10.1007/s00395-023-01028-8)
Supplement: Supplementary file 2 — Supplementary file2 (DOCX 19 KB) [file 395_2023_1028_MOESM2_ESM.docx]

Table S2. Primary antibodies used in western blots.

| Name | Manufacturer | Cat No. | Dilution | Reacts with: |
| --- | --- | --- | --- | --- |
| β-Actin | Weiao | WB0196 | 1:3000 | mice, human |
| GAPDH | Weiao | WB0197 | 1:3000 | mice, human |
| Bax | Abcam | Ab182733 | 1:1000 | mice, human |
| Bcl-2 | Abcam | Ab182858 | 1:1000 | mice, human |
| Cleaved-caspase 3 | CST | 9664T | 1:1000 | mice, human |
| Caspase 9 | Abcam | Ab202068 | 1:2000 | mice, human |
| IP3Rs | Abcam | ab108517 | 1:1000 | mice, human |
| SERCA2 | abcam | Ab150435 | 1:1000 | mice, human |
| MCU | CST | 14997S | 1:1000 | mice, human |
| CRT | abcam | Ab92516 | 1:1000 | mice, human |
| HSP90B | Abcam | Ab203085 | 1:1000 | Mice, human |
| p-eNOS Ser^1177^ | Abcam | Ab215717 | 1:1000 | mice, human |
| eNOS | CST | 32027S | 1:1000 | mice, human |
| ET-1 | Bioss | Bs-0188R | 1:500 | mice, human |
| p-VE-Cadherin Tyr^731^ | Affinity | AF3265 | 1:1000 | mice, human |
| VE-Cadherin | Thermo Fisher | 14-1441-82 | 1:1000 | mice |
| VE-Cadherin | Thermo Fisher | 14-1449-82 | 1:1000 | human |
| β-catenin | Abcam | ab32572 | 1:1000 | mice, human |
| ICAM-1 | Affinity | AF6088 | 1:1000 | mice, human |
| VCAM-1 | Abcam | Ab134047 | 1:2000 | mice, human |
| LAMP-1 | Abcam | Ab108597 | 1:2000 | human |
| LAMP-2a | Abcam | Ab125068 | 1:1000 | mice, human |
