# Supplementary material for: Pinacidil ameliorates cardiac microvascular ischemia–reperfusion injury by inhibiting chaperone-mediated autophagy of calreticulin
Source: Basic Res Cardiol. 2024 Jan 2;119(1):113–31. doi: 10.1007/s00395-023-01028-8 (PMC10837255; doi:10.1007/s00395-023-01028-8)
Supplement: Supplementary file 3 — Supplementary file3 (DOCX 17 KB) [file 395_2023_1028_MOESM3_ESM.docx]

**Table 3. Pressure-volume loop data of Sham, I/R,** **I/R+0.1mg/kg/day PIN, or I/R+0.5mg/kg/day PIN.**

|  | **Sham** | **IR** | **IR+PIN 0.1** | **IR+PIN 0.5** |
| --- | --- | --- | --- | --- |
| HR, bpm | 451±23 | 460±34 | 454±31 | 448±29 |
| ESPVR, mmHg/ml | 23.41±1.39 | 15.80±2.85* | 17.4±1.37 | 19.57±2.22# |
| EDPVR, mmHg/ml | 0.054±0.003 | 0.143±0.012* | 0.098±0.006# | 0.076±0.005# |
| Tau, ms | 7.05±0.68 | 13.63±2.76* | 9.45±2.13# | 7.94±1.98# |
| +dP/dt, mmHg/s | 6329±264 | 5334±509* | 5752±472 | 5984±505# |
| -dP/dt, mmHg/s | 7035±288 | 5694±413* | 6293±472# | 6582±516# |
| LVEDP | 6.25±0.29 | 10.23±0.56* | 8.57±0.32 | 7.74±0.48# |

HR, heart rate; ESPVR, end systolic pressure-volume relationship; EDPVR, end diastolic pressure-volume relationship; LV, left ventricle; EDP, end-diastolic pressure. Data are mean ± SEM, * p<0.05 vs Sham, # p<0.05 vs I/R using one-way ANOVA followed by Tukey test.
