# Supplementary material for: Pinacidil ameliorates cardiac microvascular ischemia–reperfusion injury by inhibiting chaperone-mediated autophagy of calreticulin
Source: Basic Res Cardiol. 2024 Jan 2;119(1):113–31. doi: 10.1007/s00395-023-01028-8 (PMC10837255; doi:10.1007/s00395-023-01028-8)
Supplement: Supplementary file 4 — Supplementary file4 (DOCX 17 KB) [file 395_2023_1028_MOESM4_ESM.docx]

**Table S4. Pressure-volume loop data of CRT overexpression.**

|  | Sham+ NC | Sham+CRT | IR+ NC | Sham+ CRT |
| --- | --- | --- | --- | --- |
| HR, bpm | 466±28 | 474±25 | 548±37* | 487±32 |
| ESPVR, mmHg/ml | 24.19±1.50 | 22.62±1.96 | 15.25±3.22* | 19.36± 2.81# |
| EDPVR, mmHg/ml | 0.058±0.004 | 0.062±0.003 | 0.135±0.008* | 0.063±0.007# |
| Tau, ms | 7.26±0.74 | 7.14±0.52 | 13.53±3.14* | 9.69±2.57# |
| +dP/dt, mmHg/s | 6837±375 | 6934±289 | 5593±654* | 6138±581# |
| -dP/dt, mmHg/s | 7034±196 | 6876±243 | 5806±539* | 6349±476# |
| LVEDP | 6.39±0.41 | 6.48±0.36 | 10.67±0.74* | 8.82±0.62# |

HR, heart rate; ESPVR, end systolic pressure-volume relationship; EDPVR, end diastolic pressure-volume relationship; LV, left ventricle; EDP, end-diastolic pressure. Data are mean ± SEM, * p<0.05 vs Sham+NC, # p<0.05 vs IR+ NC using one-way ANOVA followed by Tukey test
