# Supplementary figures and images for: Pinacidil ameliorates cardiac microvascular ischemia–reperfusion injury by inhibiting chaperone-mediated autophagy of calreticulin
Source: Basic Res Cardiol. 2024 Jan 2;119(1):113–31. doi: 10.1007/s00395-023-01028-8 (PMC10837255; doi:10.1007/s00395-023-01028-8)

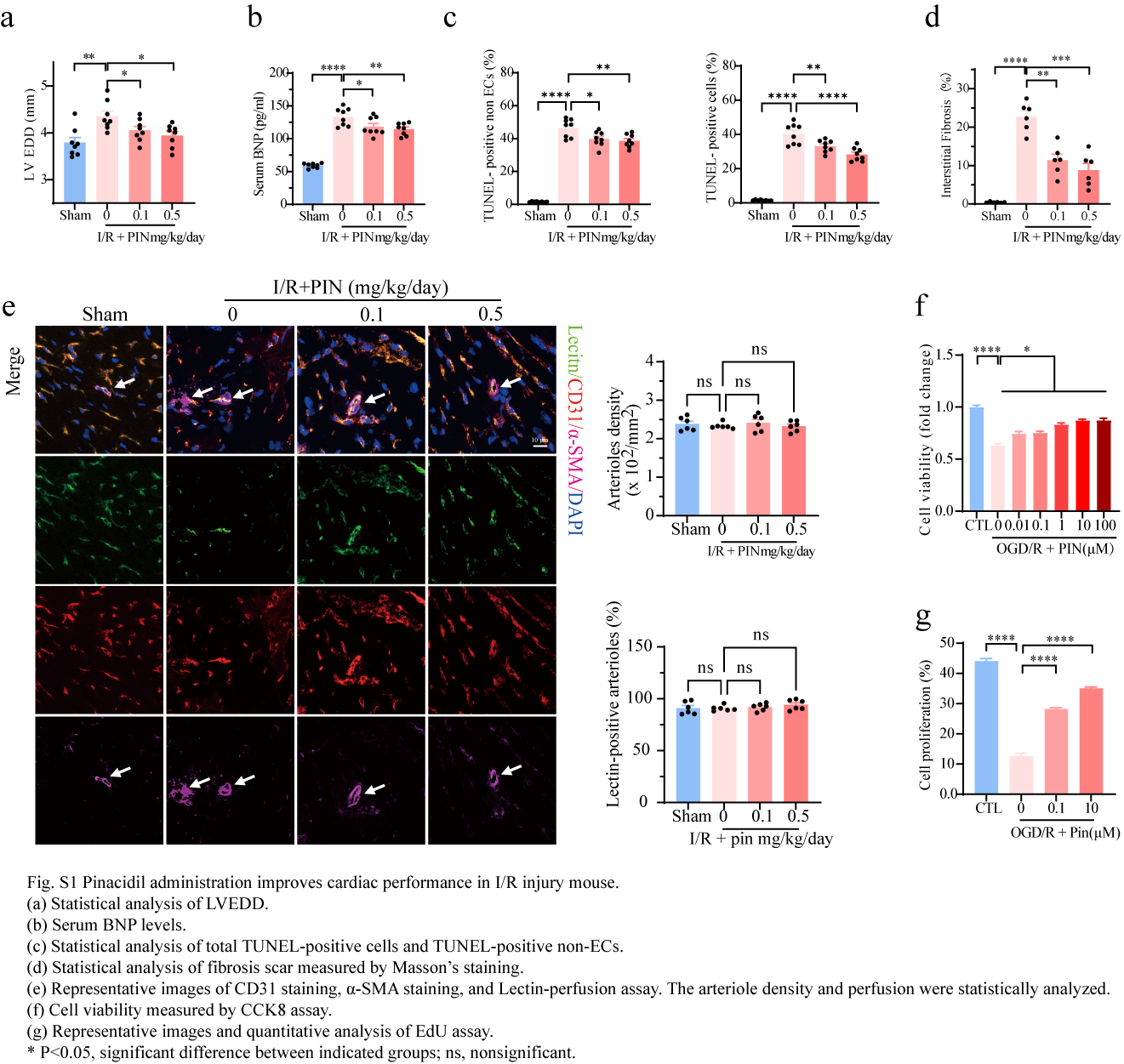

Supplement: Supplementary file 5 — Supplementary file5 (TIF 6018 KB) [file 395_2023_1028_MOESM5_ESM.tif]

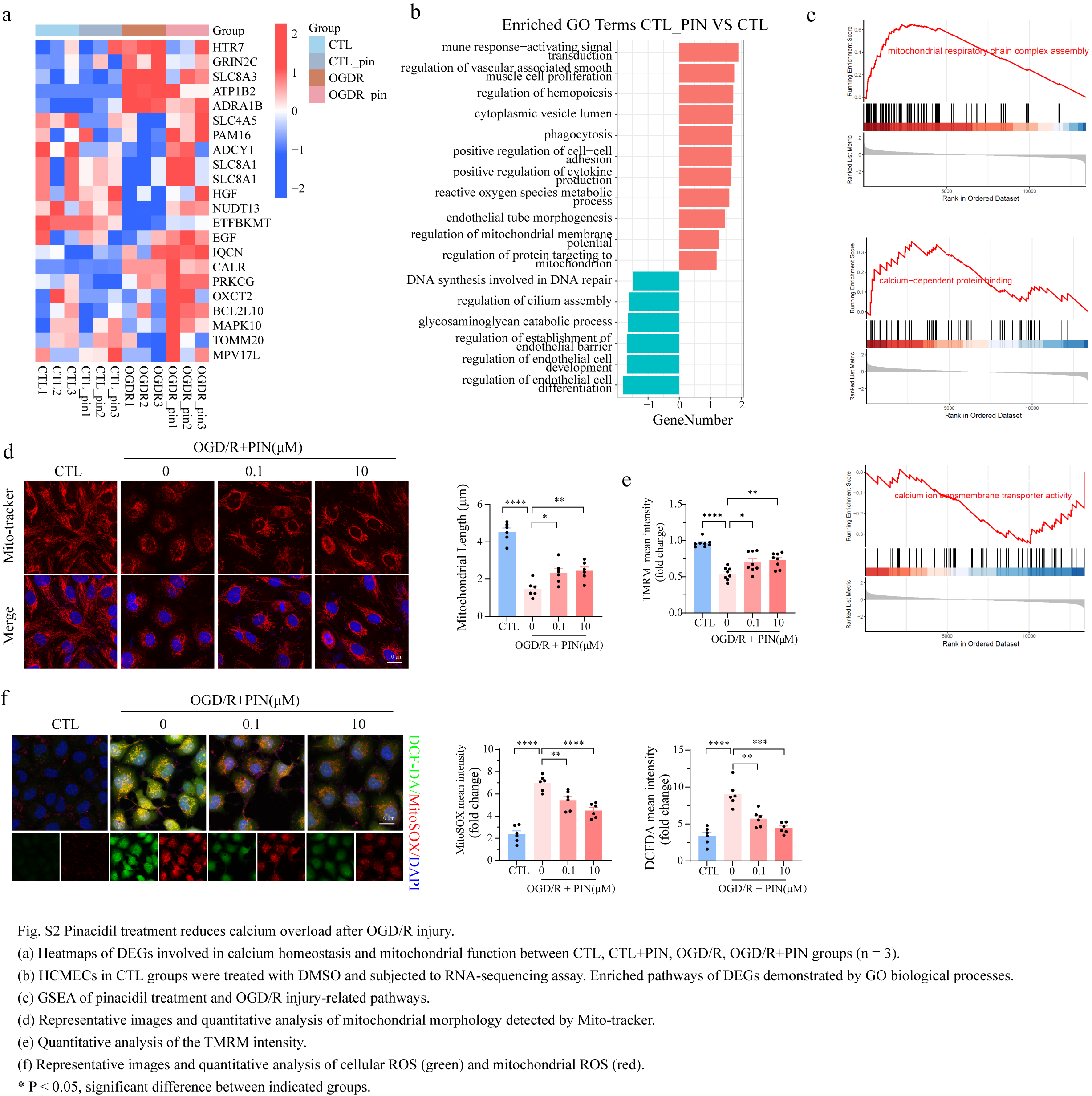

Supplement: Supplementary file 6 — Supplementary file6 (TIF 20738 KB) [file 395_2023_1028_MOESM6_ESM.tif]

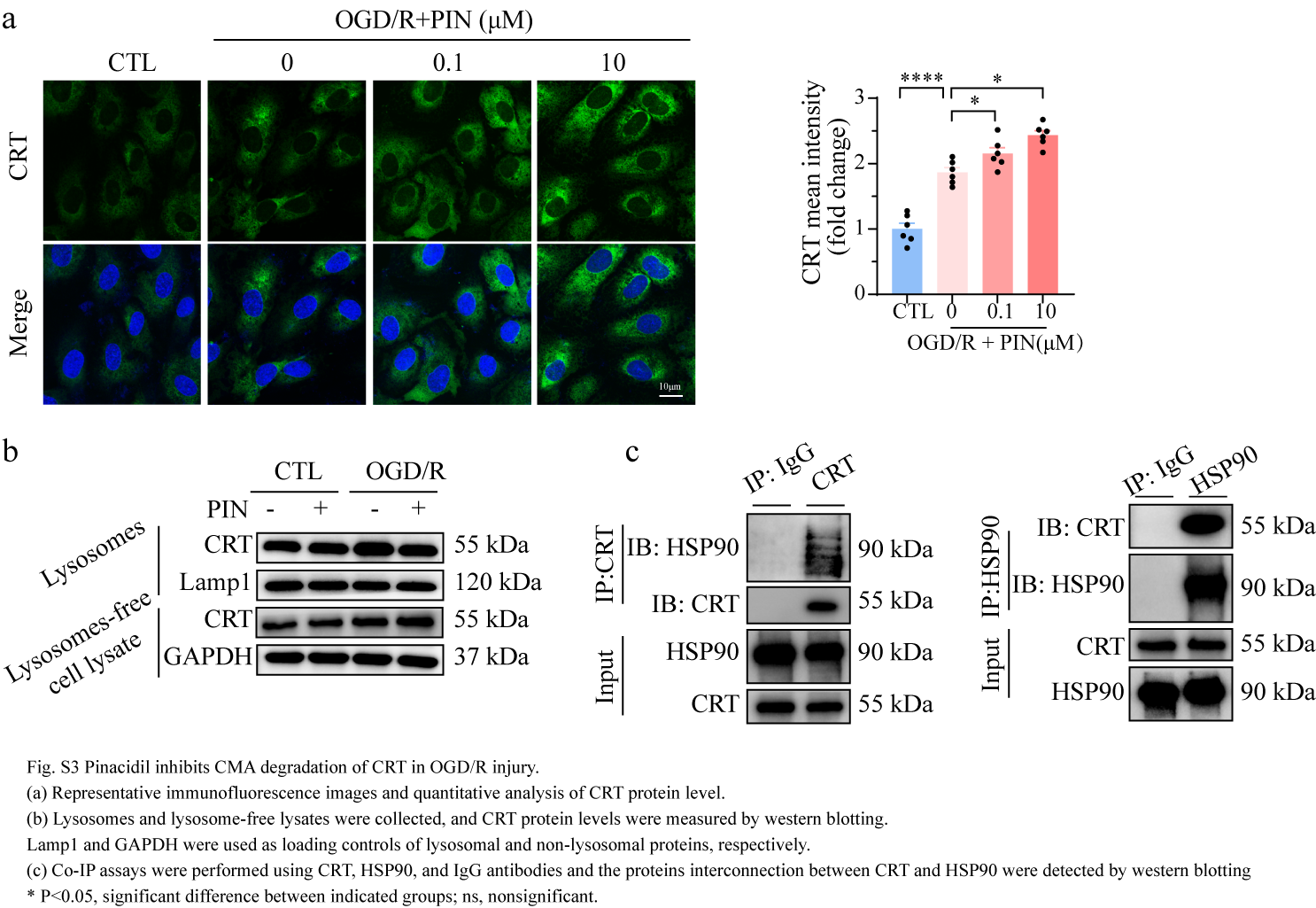

Supplement: Supplementary file 7 — Supplementary file7 (TIF 5523 KB) [file 395_2023_1028_MOESM7_ESM.tif]

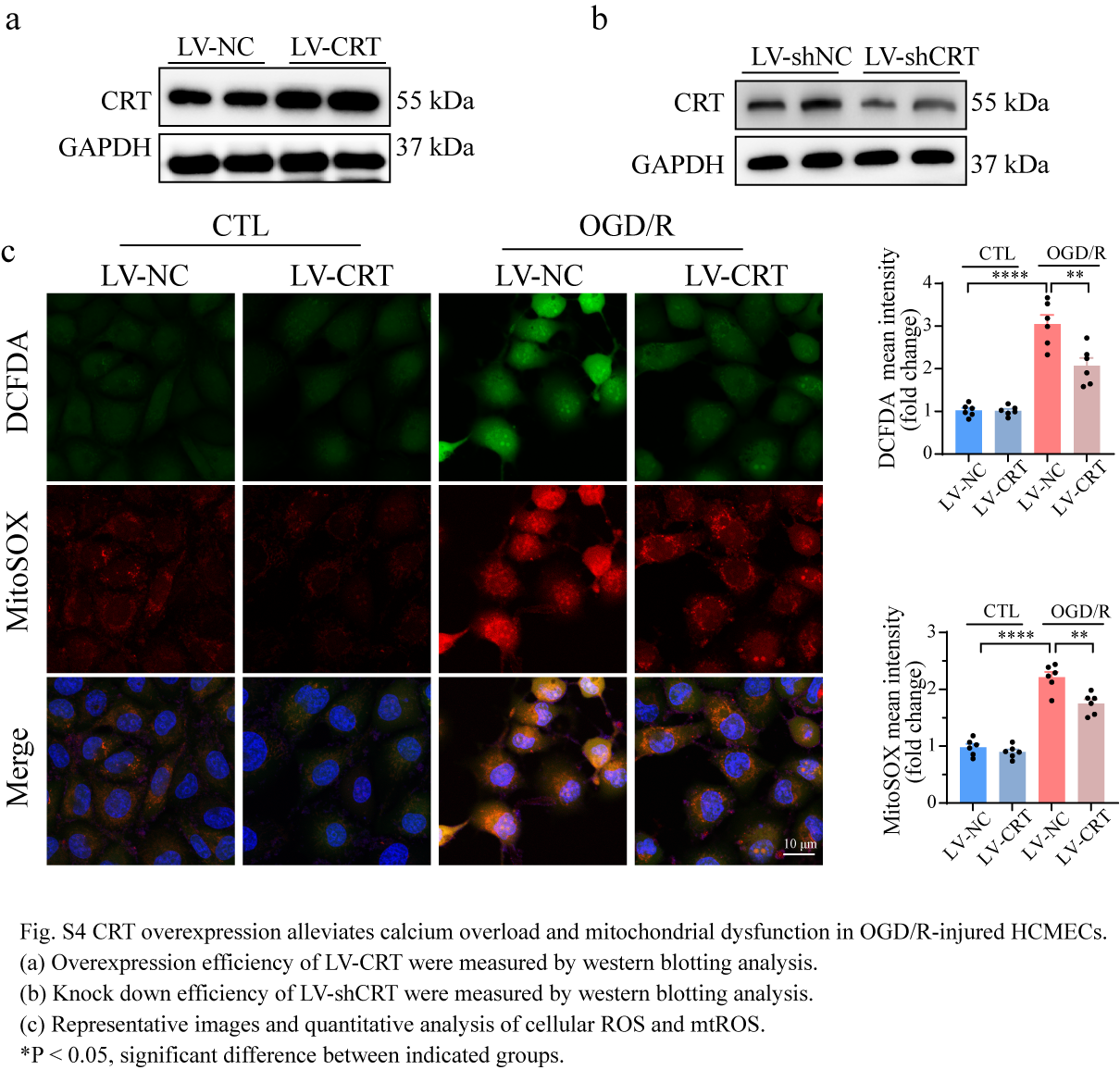

Supplement: Supplementary file 8 — Supplementary file8 (TIF 5380 KB) [file 395_2023_1028_MOESM8_ESM.tif]

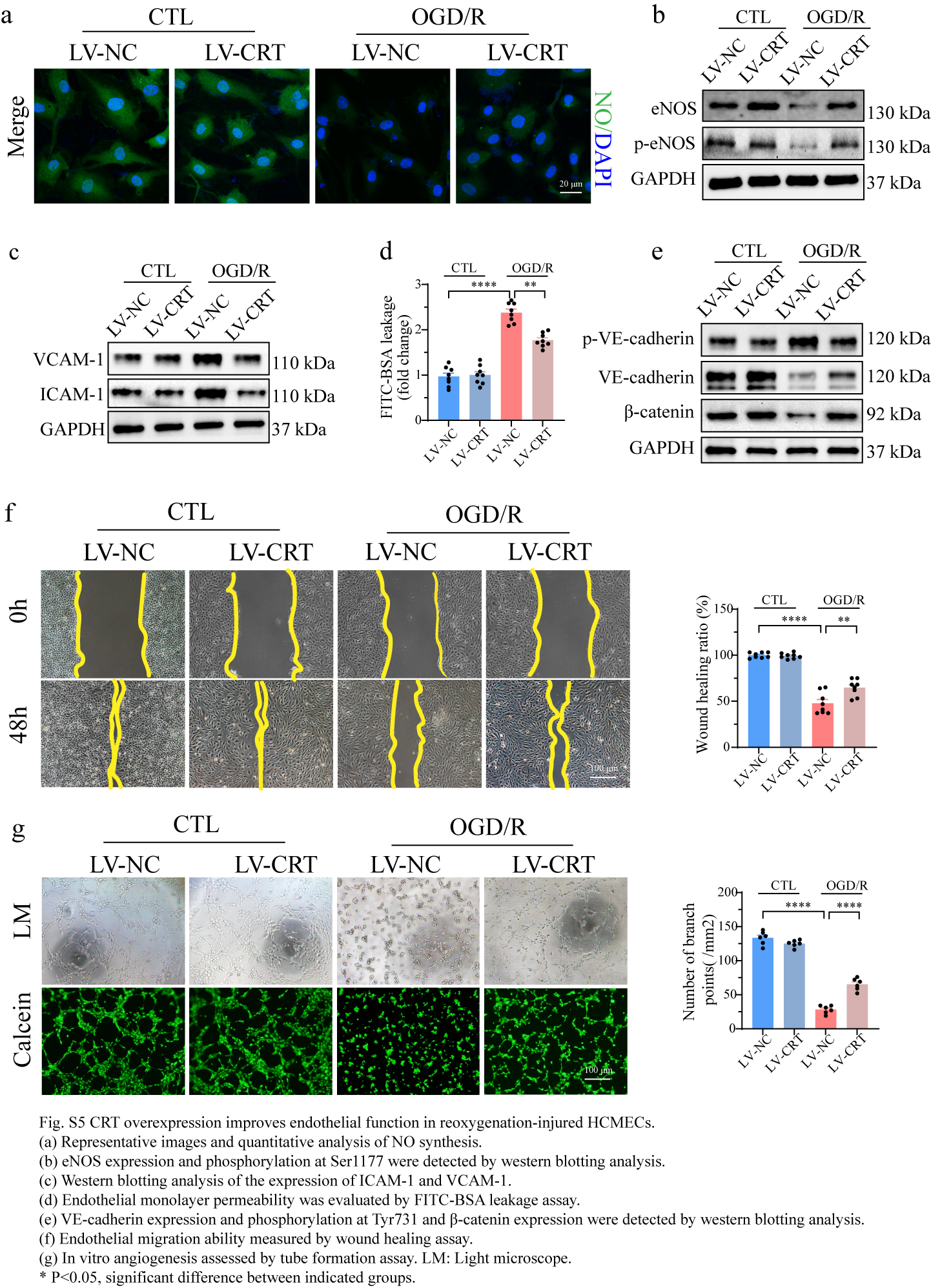

Supplement: Supplementary file 9 — Supplementary file9 (TIF 11521 KB) [file 395_2023_1028_MOESM9_ESM.tif]

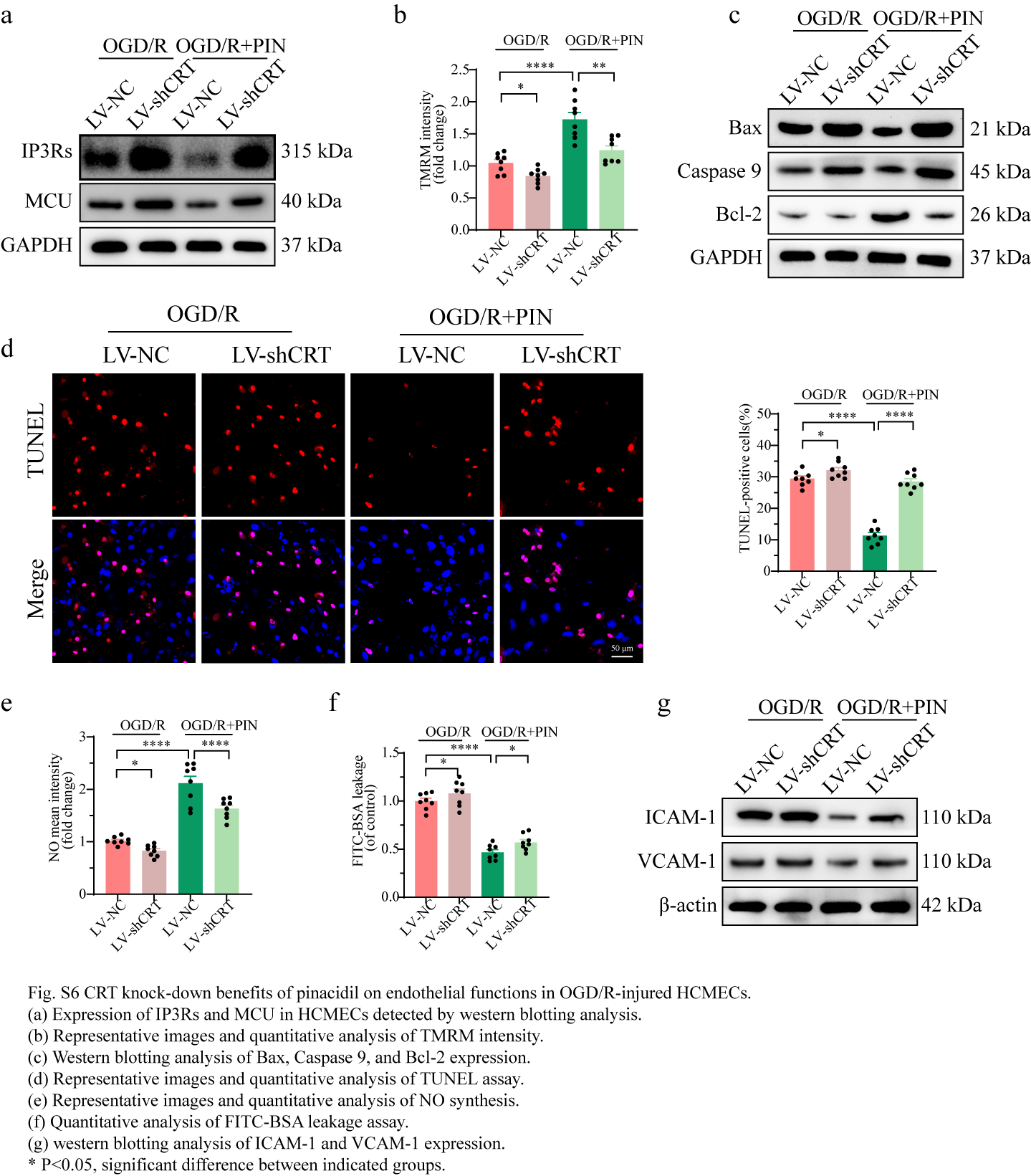

Supplement: Supplementary file 10 — Supplementary file10 (TIF 8132 KB) [file 395_2023_1028_MOESM10_ESM.tif]

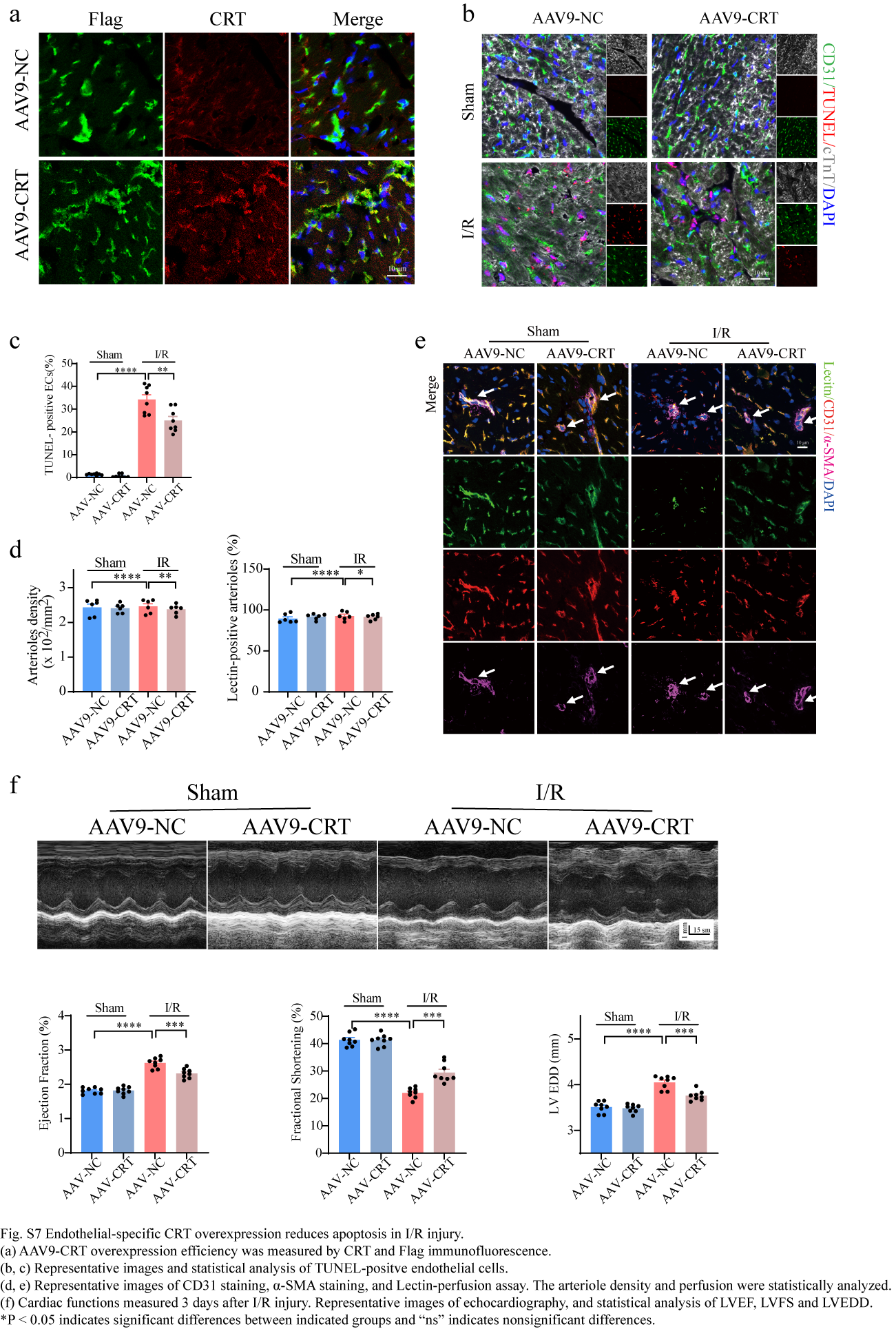

Supplement: Supplementary file 11 — Supplementary file10 (TIF 9773 KB) [file 395_2023_1028_MOESM11_ESM.tif]
